# Nanoparticulate MgH_2_ ameliorates anxiety/depression-like behaviors in a mouse model of multiple sclerosis by regulating microglial polarization and oxidative stress

**DOI:** 10.1186/s12974-023-02696-y

**Published:** 2023-01-30

**Authors:** Zhenghao Li, Kefu Chen, Qi Shao, Hongtao Lu, Xin Zhang, Yingyan Pu, Xuejun Sun, Hua He, Li Cao

**Affiliations:** 1 Institute of Neuroscience and Key Laboratory of Molecular Neurobiology of Military of Education, Naval Medical University, Shanghai, 200433 China; 2 Department of Naval Medicine, Naval Medical University, Shanghai, 200433 China; 3 Center of Hydrogen Science, Shanghai Jiao Tong University, Shanghai, 200030 China; 4 Department of Neurosurgery, Third Affiliated Hospital, Naval Medical University, Shanghai, 200438 China

**Keywords:** Multiple sclerosis, EAE, Anxiety, Depression, Microglial polarization, Neuroinflammation, MgH_2_

## Abstract

Multiple sclerosis (MS) is an autoimmune demyelinating disease of the central nervous system (CNS). Anxiety and depression are the most common psychiatric comorbidities of MS, which seriously affect patients’ quality of life, treatment compliance, and prognosis. However, current treatments for anxiety and depression in MS show low therapeutic efficacy and significant side effects. In the present study, we explored the therapeutic effects of a novel low-toxic anti-inflammatory drug, nanoparticulate magnesium hydride (MgH_2_), on mood disorders of MS. We observed that anxiety/depression-like behaviors in experimental autoimmune encephalomyelitis (EAE) mice were alleviated by MgH_2_ treatment. In addition, disease severity and inflammatory demyelination were also diminished. Furthermore, we confirmed the suppressive effect of MgH_2_ on depression in the acute restraint stress model. Mechanistically, MgH_2_ may play a therapeutic role by promoting microglial M2 polarization, inhibiting microglial M1 polarization, and reducing oxidative stress and mitochondrial damage. Therefore, nanoparticulate MgH_2_ may be a promising therapeutic drug for psychiatric comorbidities of MS.

**Supplementary Information:**

The online version contains supplementary material available at 10.1186/s12974-023-02696-y.

## Introduction

Multiple sclerosis (MS) is an autoimmune neurodegenerative disease of the central nervous system (CNS) characterized by inflammatory demyelination and axonal damage [1]. MS is the most common cause of neurological disability in young adults—aside from trauma—and usually leads to sensory-motor, emotional and cognitive impairment. Clinical treatment mostly focuses on relieving somatic dysfunction, and relatively little attention is assigned to mood disorders [2].

Anxiety and depression are the main manifestations of affective disorder and the most common psychiatric comorbidities in MS patients, the prevalence of which is rather high: 23.7% for depression and 21.9% for anxiety [3, 4]. MS comorbid with psychiatric disorders seriously affects the quality of life, disease process, and overall outcome of patients. First, anxiety and depression aggravate the physical dysfunction of MS patients and affect their ability to work, family life, and social life. Second, those with MS and mood disorders usually have cognitive dysfunction and are also less likely to be compliant with disease-modifying treatment, which may ultimately affect their MS disease course [5, 6]. Third, anxiety and depression significantly affect the prognosis by increasing mortality in people with MS. The standardized mortality rate (SMR) showed that suicide risk in MS patients was twice as high as that in the general population [7]. Fourth, more severe neurological dysfunction and poor self-care ability in MS patients might exacerbate anxious and depressive symptoms [8]. Therefore, MS comorbid with anxiety and depression needs urgent attention and effective treatment.

Currently, selective serotonin reuptake inhibitors (SSRIs) are considered first-line treatments for anxiety and depression [9, 10]. However, SSRIs commonly have limitations, such as delayed onset, low efficacy, association with metabolic syndrome, cardiovascular and gastrointestinal side effects, and sexual dysfunction. Importantly, SSRIs might increase suicidal and hostile behavior in children and adolescents with depression [11, 12]. Approximately one-third of patients with depression are resistant to existing antidepressants [13]. Moreover, current data are insufficient to determine the effectiveness of treatments for depression and anxiety in MS [14, 15]. Thus, there is an urgent need for safer and more effective drugs and new therapeutic targets.

Microglia, resident immune cells in the CNS, are important for innate and acquired immunity and play a key role in the pathogenesis of MS comorbid with anxiety and depression. Research on MS has shown that 48 MS risk genes are preferentially expressed in microglia [16]. Microglial activation in the brains of MS patients precedes immune cell infiltration in peripheral blood and demyelination of the CNS [17]. Microglia affect the progression of MS mainly through phagocytosis, inflammation regulation, antigen presentation, and synaptic pruning [18]. Clinical trials with minocycline, a microglial inhibitor, showed that the drug might impede “clinically isolated syndrome” from transitioning to MS [19]. Research on anxiety and depression has shown that microglia are activated in the brains of patients, specifically in regions related to emotion [20, 21]. Microglial activation further aggravates the severity of anxiety and depression by regulating monoaminergic and glutamatergic pathways, synaptic transmission, synaptic plasticity, hippocampal neurogenesis, and monocyte infiltration [22–25]. Adjunctive minocycline also helps to reduce anxious and depressive symptoms in clinical trials [26, 27]. Taken together, the literature suggests that anti-inflammatory therapy targeting microglia can potentially treat symptoms of anxiety and depression in MS patients. Since minocycline, a semisynthetic tetracycline-derived antibiotic, has many adverse effects, including nausea, diarrhea, dizziness, dermatitis, and dysbacteriosis [28], novel anti-inflammatory therapeutic measures with fewer side effects might have a promising future.

Nanoparticulate MgH_2_ is a novel and efficient magnesium-based hydrogen storage material that can sustainably produce massive hydrogen in the stomach through the following reaction: MgH_2_ + 2H_2_O = Mg(OH)_2_ + 2H_2_. H_2_ is a biologically inert gas that is easy to use and has hardly any side effects [29]. Increasing evidence has shown that H_2_ suppresses neuroinflammation and oxidative stress, which are major contributors to anxious and depressive disorders. Indeed, it is reported that hydrogen-rich water treatment prevents depressive-like behavior in animal model of stress [30]. Compared with the traditional administration of H_2_ such as hydrogen-rich water, MgH_2_ not only releases more H_2_ sustainably but also supplies magnesium simultaneously. As the second most abundant cation in mammalian cells, magnesium is involved in numerous important biological processes. Magnesium is a co-enzyme for energy production in the mitochondria [31]. In addition, it has anti-inflammatory effects and inhibits ROS production in the brain [32–34]. Importantly, magnesium plays a vital modulatory role in brain biochemistry, influencing several neurotransmission pathways associated with the development of anxiety and depression [35]. Intravenous and oral magnesium has been reported to rapidly terminate depressive symptoms safely and without side effects [36]. However, there have been no studies investigating the function and mechanism of MgH_2_ in MS comorbid with anxiety and depression.

In this study, we explored the feasibility of MgH_2_ as a novel, safe, and effective drug for relieving psychiatric symptoms of MS. Our study found that MgH_2_ can reduce neuroinflammation in mouse model of experimental autoimmune encephalomyelitis (EAE) and acute restraint stress model, thereby alleviating demyelination-induced motor dysfunction and anxiety/depression-like symptoms. However, MgH_2_ has no effect on remyelination in the lysophosphatidylcholine (LPC) model. We hypothesize that MgH_2_ exerts its effects by regulating microglial polarization and inhibiting oxidative stress.

## Materials and methods

### Animals

All animal experiments were performed in adherence with the National Institutes of Health Guide for the Care and Use of Laboratory Animals and approved by the Naval Medical University Committee on Animal Care. C57BL/6 mice were purchased from Shanghai SLAC Laboratory Animal Co., Ltd (Shanghai, China), maintained under specific pathogen-free conditions, and used at 8–10 weeks of age. We anesthetized mice with pentobarbital, and then the whole brain and spinal cords were collected following transcardial perfusion and overnight post-fixation with 4% paraformaldehyde.

### Experimental autoimmune encephalomyelitis (EAE) model

The EAE model was induced by myelin oligodendrocytes glycoprotein (MOG35–55) as previously described [37]. Briefly, female C57BL/6 mice (8 weeks) were subcutaneously immunized with MOG 35–55 (GL Biochem, Shanghai, China) in complete Freund’s adjuvant containing heat-killed Mycobacterium tuberculosis (H37Ra strain; Difco), Pertussis toxin (516561, Calbiochem–EMD Chemicals, San Diego, CA) in PBS was administered intraperitoneally (i.p.) 0-day (d) post-injection (dpi) and 2 dpi. MgH_2_ was administered at 1 dpi. Clinical EAE scores on a scale of 0–5 were examined daily in a blind manner as follows: 0, no clinical signs; 1, paralyzed tail; 2, paresis; 3, paraplegia; 4, paraplegia with forelimb weakness or paralysis; and 5, moribund state or death.

### The procedure of the 24-h restraint

The 24-h restraint of mice was conducted as previously described [38] and we modified it partially. Briefly, mice were placed in 50 mL centrifuge tubes and subjected to 24 h restraint from 10:00 a.m. to 10:00 a.m. of the next day. The holes (~ 0.5 cm in diameter) in the head, tail, and side wall of 50 mL centrifuge tubes made airflow. Mice could move their head and anterior limb, but the body and hindquarters were not able to move or turn around. During the restraint, mice had no access to food and water. Once the restraint ended, mice were immediately put back into their home cages, with free access to food and water. Non-restraint mice (control group) remained in the home cages and also had no access to food and water for 24 h. Mice in the experimental group were immediately fed with MgH_2_ after restraint and this continued until the end of the behavioral experiments.

### Lysophosphatidylcholine (LPC) model

Adult (8–10 weeks) female C57BL/6 mice were deeply anesthetized with 1.5% isoflurane in 30% oxygen. Focal demyelination in the dorsal spinal cord was induced by LPC (Sigma-Aldrich, Catalog#62962) as described previously [39]. Briefly, 1 μL of LPC (0.1% in saline) was injected into the dorsal column at the T11–T12 vertebrae with a micromanipulator. MgH_2_ was administered at 1 dpi. Mice were anesthetized and sacrificed at 7 dpi and 14 dpi after LPC injection, then the spinal cord containing the injection lesion was collected and cut into serial paraffin sections. The demyelinated lesion volume was calculated based on the equation: V = Σ demyelinated lesion area x thickness of the section.

### Drug administration

For the treatment of EAE mice, 24-h restraint mice, and LPC mice, we fed the experimental group with 0.5% MgH_2_ (AIN93G + 0.5% MgH_2_), and the control group was fed with standardized feed (AIN93G). To determine the concentration of MgH_2_ in vitro, we detected the effect of MgH_2_ on the viability of BV2 cells (Additional file 1: Fig. S1). MgH_2_ powder (obtained from the Center of Hydrogen Science, Shanghai Jiao Tong University) was suspended in propylene glycol, and for cell experiments, the control group was added with the same dose of propylene glycol in vitro*.*

### Behavioral procedures

Before each experiment, mice were put into the behavioral laboratory for 1–2 h in advance. All behavioral experiments were performed between 9:00 a.m. and 5:00 p.m. Behaviors were recorded, stored, and analyzed using an automated behavioral tracking system (Smart 3.0.06, Panlab Harvard Apparatus) equipped with infrared lighting-sensitive CCD cameras.

*Open field test* Mice were placed in the corner of an open-field apparatus (40 cm × 40 cm) for 10 min of spontaneous exploration with an overhead video-tracking system. The total distance mice traveled, the distance they traveled in the center area (20 cm × 20 cm center square), and the time they spent and entries in the center area were monitored throughout the experiment.

*Marble burying test* The marble burying test was performed on mice as previously described with minor modifications [40]. Briefly, mice were placed individually in an open arena (50 cm × 50 cm × 30 cm) containing fresh bedding (5 cm deep) with 20 clean marbles prearranged in a 5 by 4 grid. Mice were allowed to bury the marbles for 20 min. After the test period, buried marbles were counted manually. Marbles were considered buried if they were at least 2/3 covered with bedding.

*Light/dark test* The light/dark test (LDT) is based on the innate aversion of rodents to brightly lit areas [41]. LDT was performed as previously described with minor modifications [42]. Mice were gently placed in a cage (25 cm × 25 cm × 25 cm) divided into a dark chamber and an illuminated chamber by a partition with a door. The mice were allowed to move freely between the two chambers with the door open for 10 min. Video tracking data were analyzed using software to extract the movement trail, the time spent in each chamber, and transition numbers.

*Sucrose preference test* The sucrose preference test was performed using a two-bottle procedure, during which mice had free access to both water and a sucrose solution, as previously described [43], with minor modifications. First, mice need to habituate to consuming water from the two bottles 2 d in advance. After habituation, mice were deprived of water and the sucrose preference was measured during the next 3 d. The first 2 days served as habituation to sucrose solutions. The results of day 3 were used for the evaluation of sucrose preference. Each day, group-housed mice were placed individually into small plastic cages and were presented for 12 h with two bottles—one with tap water and one with a 2% sucrose solution. Consumption of water or sucrose solution was measured by weighing the volume of two bottles. Bottles were counterbalanced across the left and the right sides of the cage, and their position was alternated from test to test. Sucrose preference (percent) was calculated as follows: Preference = [sucrose solution intake (mL)/total fluid intake (mL)] × 100.

*Tail suspension test* The tail suspension test was performed based on the previously described [42]. Mice were suspended by their tails with tape in a position that they could not escape or hold on to nearby surfaces for 6 min. Immobility was quantified in the last 4 min of the test using the Video track system.

*Forced swimming test* The forced swimming test was performed as previously described [44]. Mice were placed in a glass beaker (24 cm tall, 14 cm diameter) containing 15 cm deep water at 24 ± 1 °C. Mice were allowed to swim for 6 min. Immobility was quantified in the last 4 min of the test using the Video track system.

### Cells cultures

Detailed methods for the primary culture and purification of microglia and OPCs were previously described [45]. Briefly, Postnatal day 1 (P1) C57BL/6 mice were sacrificed to acquire mixed cerebrum glial cell cultures containing microglia. After 10–14 days of growth in DMEM/F12 medium (Invitrogen) containing 10% fetal bovine serum (FBS, GBICO), the cultures were shaken for 1 h (180 rpm, 37 °C) to collect the microglia. For M1 polarization, the microglia were stimulated with lipopolysaccharide (LPS, 10 ng/ml, R&D). For M2 polarization, the microglia were stimulated with IL-4 (10 ng/ml, R&D).

To obtain OPCs, P1 Sprague Dawley rats (SD rats) were sacrificed to acquire mixed cortical glial cell cultures. After 8–10 days of growth in DMEM containing 10% FBS, the cultures were shaken for 1 h (180 rpm, 37 °C) to remove the microglia. After replacing the medium, the cultures were shaken for another 14 h (200 rpm, 37 °C) to collect the OPCs. OPCs were cultured in cultured DMEM/F12 medium supplemented with PDGFaa (0.1%), B27 (2%), N2 (1%) and BFGF (0.1%) for proliferation, and cultured in neurobasal medium supplemented with 2% B27 for differentiation. 10 ng/ml thyroid-hormone (T3, Sigma) was used as a positive control for differentiated OPCs.

### RNA isolation and quantitative real-time PCR (qPCR)

Total RNA was extracted from the primary cell culture using Trizol (Invitrogen, Carlsbad, USA). First-strand cDNA was synthesized using a RevertAid First Strand cDNA Synthesis kit (Thermo Fisher Scientific, Waltham, USA). qPCR was performed on a LightCycler 96 apparatus (Roche) using the SYBR Green Real-time PCR Master Mix (TOYOBO, Shanghai, China). Gene expression was expressed as the mRNA level, which was normalized to that of a standard housekeeping gene (Gapdh) using the △△CT method. The primer pairs were as follows: for *inos*, F: TTGACGCTCGGAACTGTAG; R: GACCTGATGTTGCCATTGT; for *Tnf-α*, F: GCCTCCCTCTCATCAGTTCT; R: ACTTGGTGGTTTGCTACGAC; for *Arg-1*, F: GCTTGCTTCGGAACTCAAC; R: CGCATTCACAGTCACTTAGG; for *Ym1*, F: TACTCCTCAGAACCGTCAGAT; R: CATTTCCTTCACCAGAACAC; for *Fizz1*, F: ATGCCAACTTTGAATAGGATG; R: CTTGACCTTATTCTCCACGAT; for *Gapdh*, F: TCAACGACCCCTTCATTGACC; R: CTTCCCGTTGATGACAAGCTTC.

### Western blot analysis

Primary cell cultures were homogenized in RIPA buffer that was supplemented with protease cocktail inhibitors (Beyotime, Shanghai, China). Cell lysates were subjected to Western blot analysis using anti-inducible nitric oxide (iNOS; 1: 500, Cell Signaling Technology, Catalog#13120), anti-Arginase-1 (1: 1000, Santa Cruz Biotechnology, Catalog#sc-271430), anti-MBP (1: 500, MilliporeSigma, Catalog#MAB382), anti-MAG (1: 500, Santa Cruz Biotechnology, Catalog#C0217). The protein bands were analyzed and quantified using Image Lab (ODYSSEY CLX, LI-COR, America), normalizing target proteins to GAPDH (1:10000, proteintech, Catalog#10494) bands.

### Immunofluorescence staining

Cells or tissue sections were fixed, permeabilized, and incubated with primary antibodies anti-Sox10 (1: 200, R&D Systems, Catalog#AF2864), anti-CC1 (1: 200, Millipore, Catalog#OP80), anti-Iba1 (1: 200, Abcam, Catalog#ab48004), anti-GFAP (1: 200, Abcam, Catalog#ab4674), anti-Olig2 (1: 200, Millipore, Catalog#ab9610), anti-MBP (1: 50, Abcam, Catalog#ab7349), anti-NF200 (1: 200, MilliporeSigma, Catalog#N4142), anti-Neurofilament-H (NF-H), nonphosphorylated (clone SMI32, 1: 400, Biolegend, Catalog#801701), anti-PDGFRα (1: 200, R&D Systems, Catalog#AF1062), anti-Ki67 (1: 300, Cell Signaling Technology, Catalog#9129) and anti-Caspase3 (1: 100, Millipore, Catalog#AB3623) overnight at 4 °C, followed by incubation with TRITC-conjugated, FITC-conjugated secondary antibody, or Alexa647-conjugated secondary antibody (1: 200, Jackson ImmunoResearch) and counterstained with Hoechst33342 (1: 1000, Sigma-Aldrich) for 2 h at room temperature. Fluorescence images were captured using fluorescence microscopy (Dragonfly 200, ANDOR, England) and quantified using Image-Pro Plus (Media Cybernetics).

### Luxol fast blue (LFB) and histological staining

The brains or spinal cords were isolated from EAE and restraint mice and cut into continuous paraffin sections (4 μm). LFB kit (Servicebio, G1030) was used for LFB staining. For hematoxylin and eosin (H&E) staining, sections were immersed in hydrochloric acid-alcohol for several seconds followed by 1% ammonia water for several seconds, and then placed into Iraqi red dye for 1–3 min. Finally, the slices were dehydrated with ethanol for 5 min and made transparent with xylene for 5 min.

### Measurement of ROS generation

Reactive oxygen species assay kit (Beyotime, Shanghai, China) was used to detect the intracellular generation of ROS. The cells were incubated in serum-free media with 10 μmol/L 2,7-Dichlorodihydrofluorescein diacetates (DCFH-DA) at 37 °C for 20 min. Next, the cells were washed three times with a serum-free medium. Samples were analyzed at an excitation wavelength of 488 nm and an emission wavelength of 525 nm using a flow cytometer (Beckman Coulter, Kraemer Boulevard Brea, CA, USA).

### Mitochondrial membrane potential measurement

The changes in mitochondrial membrane potential (MMP) were measured with an MMP assay kit with JC-1 (MCE, HY-K0601, Monmouth Junction, NJ, USA) according to the manufacturer’s instructions. Briefly, treated cells were harvested and washed with cold PBS one time. Then, the cells were suspended in a mixture of 200 μL of culture medium and 1 μL of JC-1 staining solution for 20 min in the dark at 37 °C. Subsequently, the cells were washed with cold staining buffer three times. JC-1 existed either as a cytoplasmic JC-1 monomer or in mitochondrial J-aggregates, depending on the MMP. After the corresponding treatment, the cells were stained with JC-1 as described above and visualized by fluorescence microscopy (N1-E, Nikon, Japan). Depolarization of MMP is measured in terms of increased fluorescence of JC-1 monomers (green) relative to its aggregates (red).

### BrdU incorporation

For animals, BrdU (5-bromo-2-deoxyuridine, Sigma) was intraperitoneally injected with a dose of 100 mg/kg (body weight) after the 24-h restraint once a day for 7 consecutive days. For the cell experiment, 10 μmol/L BrdU was added to the medium and incubated for 6 h to label proliferating cells. After fixation in 4% PFA, cells were rinsed three times with 1 × PBS for 5 min, permeabilized with 0.3% Triton X-100 for 10 min, then incubated in 2 N HCl for 30 min and neutralized in 0.1 mol/L sodium borate for 25 min. Finally, the cells were incubated with primary anti-BrdU (1: 100, Sigma-Aldrich) overnight at 4 °C as described for immunocytofluorescence staining above.

### Statistical analysis

The data were analyzed using One-way ANOVA with Fisher LSD test or Dunnett’s multiple comparisons test for pairwise comparisons in multiple groups (for variables with homogenous variance) and Games–Howell (for variables with non-homogenous variance). Student’s *t* test or non-parametric test of Mann–Whitney *U* was used for two groups. The data are presented as mean ± SEM. The value of *P* < 0.05 was considered statistically significant. All statistical analyses were made using Prism 6 (GraphPad) and PASW Statistics 18 (SPSS, IBM).

## Results

### MgH_2_ treatment attenuates EAE progression and inflammation-induced demyelination

**Fig. 1:**
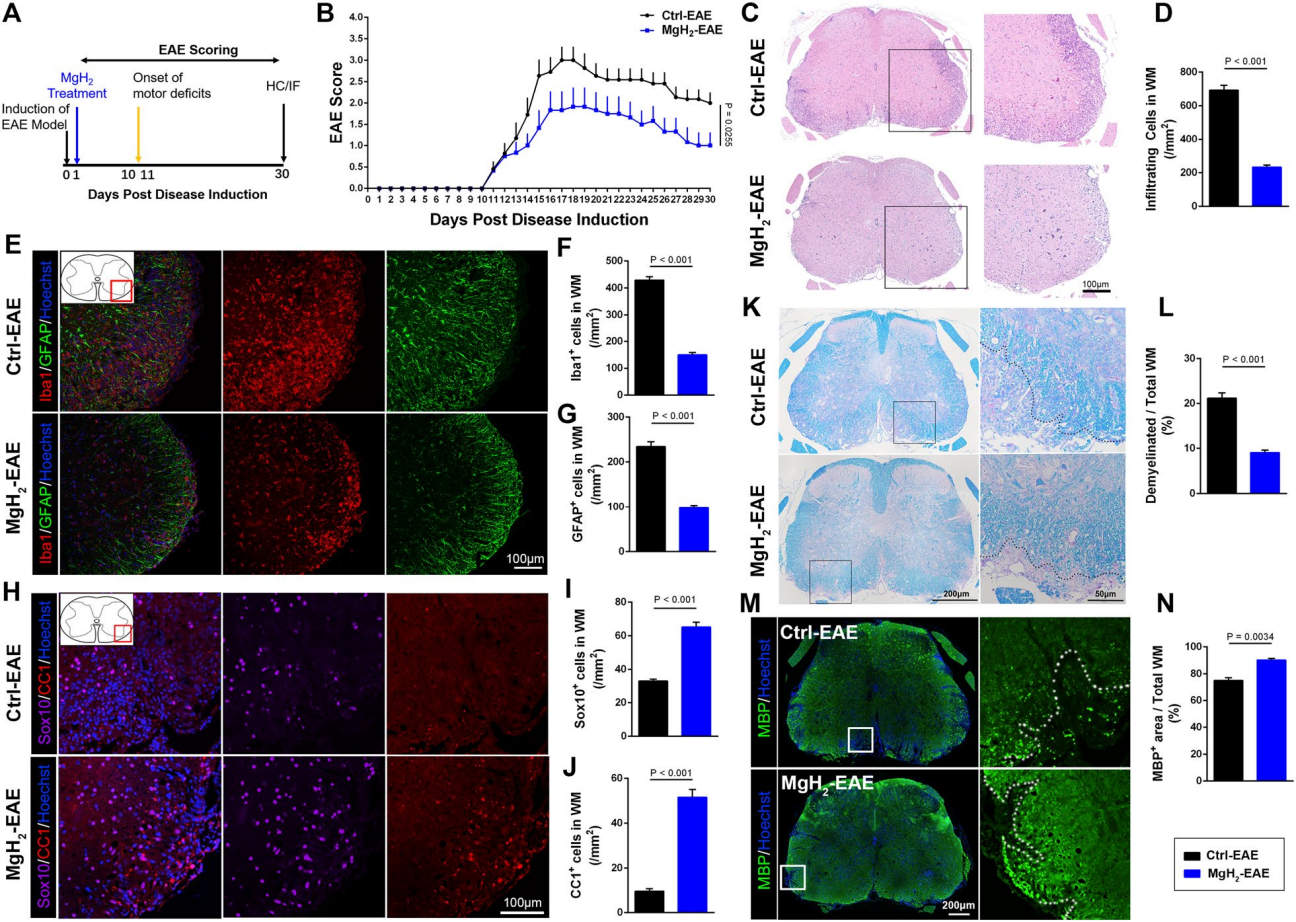
MgH_2_ treatment alleviates the progression of EAE. **A** Schematic diagram displaying the time course of EAE progression and interventions. **B** Average clinical score of EAE in mice of the control and MgH2-treated groups. *n* > 11 mice per group. **C, D** Representative spinal cord sections of H&E staining from EAE mice at 30 dpi and quantitative analysis of the number of infiltrating cells around lesion sites in WM of EAE mice (*F* = 2.6502). Infiltrating cells refer to the increased number of cells in the lesion area compared to the normal area. *n* = 3 mice per group. **E–G** Representative anti-Iba1 (red) and anti-GFAP (green) immunofluorescence of spinal cord sections around lesion sites of EAE mice at 30 dpi and quantitative analysis of Iba1^+^ cells/mm^2^ (*F* = 0.2619) and GFAP^+^ cells/mm^2^ (*F* = 3.5610), *n* = 3 mice per group. **H–J** Representative anti-CC1 (red) and anti-Sox10 (purple) immunofluorescence of spinal cord sections around lesion sites of EAE mice at 30 dpi and quantitative analysis of Sox10^+^ cells/mm^2^ (*F* = 3.7464) and CC1^+^ cells/mm^2^ (*F* = 1.8978), *n* = 3 mice per group. **K, L** Representative spinal cord sections of Luxol fast blue (LFB) staining of EAE mice at 30 dpi and quantitative analysis of the percentage of demyelinated WM in total WM (*F* = 1.0580). *n* = 3 mice per group. **M, N** Representative anti-MBP (green) immunofluorescence of spinal cord sections of EAE mice at 30 dpi and quantitative analysis of MBP^+^ area (*F* = 1.7836). *n* = 3 mice per group. Data are presented as mean ± SEM

The EAE model, which can cause neurodegenerative inflammatory disease, is often used to simulate multiple sclerosis in mice. To test whether MgH_2_ is effective for EAE, the mice were fed a standardized diet containing 0.5% MgH_2_ (AIN93G + 0.5% MgH_2_) from the induction of the model until 30 day post-injection (dpi) (Fig. 1A). We recorded the clinical scores of mice daily and found that the mean clinical scores of EAE in MgH_2_-treated mice were lower than those of the control-EAE group (Fig. 1B). In terms of the incidence of EAE, 9 of the 12 mice in the MgH_2_-treated group had symptoms, while all 11 mice in the control group had symptoms (data not shown).

Inflammation-induced demyelination is the core pathological feature of the EAE model. In this study, H&E staining showed a significant difference in the number of inflammatory cells between the MgH_2_-EAE and control-EAE mice. The infiltration of inflammatory cells in the ventral lumbar spinal cord at 30 dpi was higher in the control mice compared to the MgH_2_-treated mice (Fig. 1C, D). To assess the changes in specific types of glia in the white matter (WM) of the spinal cord, we performed immunofluorescence staining for Ionized calcium-binding adaptor protein-1 (Iba1), Glial fibrillary acidic protein (GFAP), SRY-Box Transcription Factor 10 (Sox10) and adenomatous polyposis coli (APC aka CC1). At 30 dpi, more Iba1^+^ and GFAP^+^ cells accumulated in the lesions of control-EAE mice (Fig. 1E–G), suggesting that microglia and astrocytes are significantly activated compared with the MgH_2_-EAE group. In addition, Sox10^+^ oligodendrocyte lineage cells and CC1^+^ cells (differentiated oligodendrocytes) were higher within lesions of MgH_2_-EAE mice (Fig. 1H–J). In addition, Luxol fast blue (LFB) stained a lower proportion of demyelinated area in MgH_2_-EAE mice (Fig. 1K, L). A higher proportion of the WM area stained positive for myelin basic protein (MBP) in the MgH_2_-EAE mice (Fig. 1M, N), indicating that demyelination was milder in the MgH_2_-EAE group. Together, our findings demonstrate that MgH_2_ treatment can attenuate EAE progression by alleviating inflammation and demyelination of the spinal cord in EAE mice.

### MgH_2_ treatment relieves anxiety/depression-like behaviors in EAE mice

**Fig. 2:**
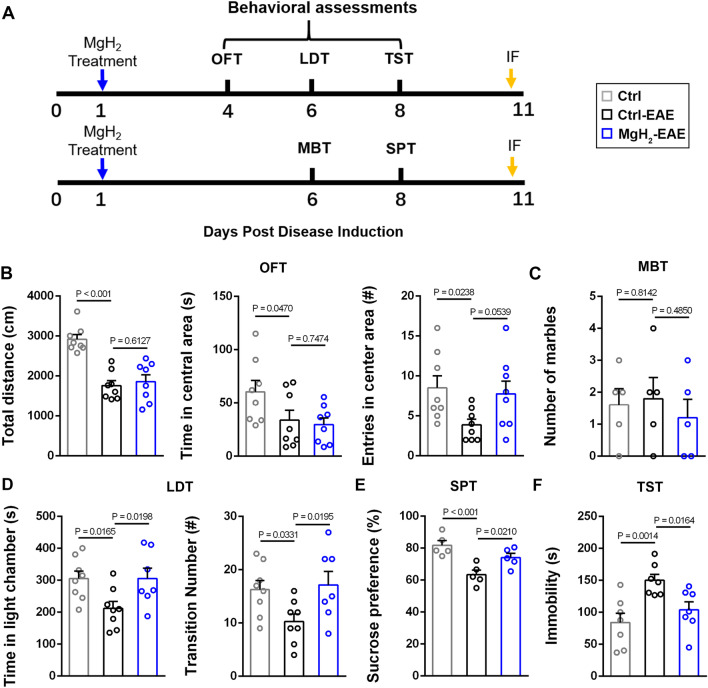
Behavioral changes occur in EAE mice and can be improved by MgH_2_. **A** Schematic diagram displaying the behavioral test protocol used in the study. **B** Open field test (OFT), statistical analysis of total distance (*F* = 20.9005), time (*F* = 3.4964) and entries (*F* = 3.4233) in the central area, *n* = 8 mice per group. **C** Marble burying test (MBT), statistical analysis of the number of buried marbles (*F* = 0.2692), *n* = 5 mice per group. **D** Light/dark transition test (LDT), statistical analysis of time in the light chamber (*F* = 4.4714) and transition numbers (*F* = 3.9609), *n* > 7 mice per group. **E** Sucrose preference test (SPT), statistical analysis of the percentage of sucrose consumption (*F* = 10.4649), *n* = 5 mice per group. **F** Tail suspension test (TST), statistical analysis of immobile time (*F* = 7.5058), *n* = 7 mice per group. Data are presented as mean ± SEM

EAE progression is usually divided into three phases: pre-onset, onset, and disease. In the pre-onset stage (0–10 dpi), mice with EAE do not show any obvious motor deficits. At the onset of EAE progression (10–12 dpi), paralyzed tails mark the beginning of motor defects. During the disease phase (12–30 dpi), mice with EAE develop more serious motor dysfunctions, including paresis, paraplegia, paralysis, and even death [46]. We observed similar phases of EAE progression in our study (Fig. 1B). Since the obvious motor dysfunction possibly affects the assessments of anxiety and depression, behavioral experiments of emotional changes were performed during the pre-onset phase (Fig. 2A).

To demonstrate whether MgH_2_ treatment alleviated emotional impairment after EAE, several behavioral tests were used. The open field test (OFT) has been used to assess anxiety behavior during EAE [46] and can also assess locomotor function. We found a significant difference in the total distance between the control group (without EAE nor MgH_2_ treatment) and the control-EAE group (without MgH_2_ treatment), indicating that the EAE modeling reduced the movement of mice (Fig. 2B). There was no significant difference in the total distance traveled by the control-EAE group and MgH_2_-EAE group (Fig. 2B). The time spent and the number of entries in the central area of the open field are used to measure anxiety-like, depressive, and exploratory behavior. Anxious and depressed mice prefer to stay in the corner, hence their time and entries in the central area decrease markedly. The control-EAE group showed fewer time and entries compared with the control group (Fig. 2B), suggesting that the EAE modeling induced an anxiety/depression-like behavior. However, the MgH_2_-EAE group showed similar time in the center but more entries (Fig. 2B), indicating that the anxiety/depression-like behavior was improved mildly by MgH_2_.

Next, we assessed the anxiety-like behavior using the marble burying test (MBT) and the light/dark transition test (LDT). Although there were no significant differences in MBT among the different treatment groups (Fig. 2C), significant differences were found in the LDT, specifically in the time spent in the light chamber and the number of transitions (Fig. 2D). Similarly, the LDT results suggest that MgH_2_ alleviates the anxiety-like behavior due to EAE modeling.

Moreover, the sucrose preference test (SPT) and the tail suspension test (TST) were applied to test the antidepressant-like effect of MgH_2_. The SPT and TST results suggested that the control-EAE group had depression-like responses, as the control-EAE mice, compared with the control mice, consumed a lower relative percentage of sucrose and were immobile longer (Fig. 2E, F). However, MgH_2_ treatment increased the sucrose preference and decreased the immobile time (Fig. 2E, F).

Overall, these behavioral data indicate that the anxiety/depression-like behaviors following EAE can be alleviated by MgH_2_ treatment.

### MgH_2_ treatment alleviates inflammation, demyelination, and axon damage in the hippocampus and corpus callosum after EAE

**Fig. 3:**
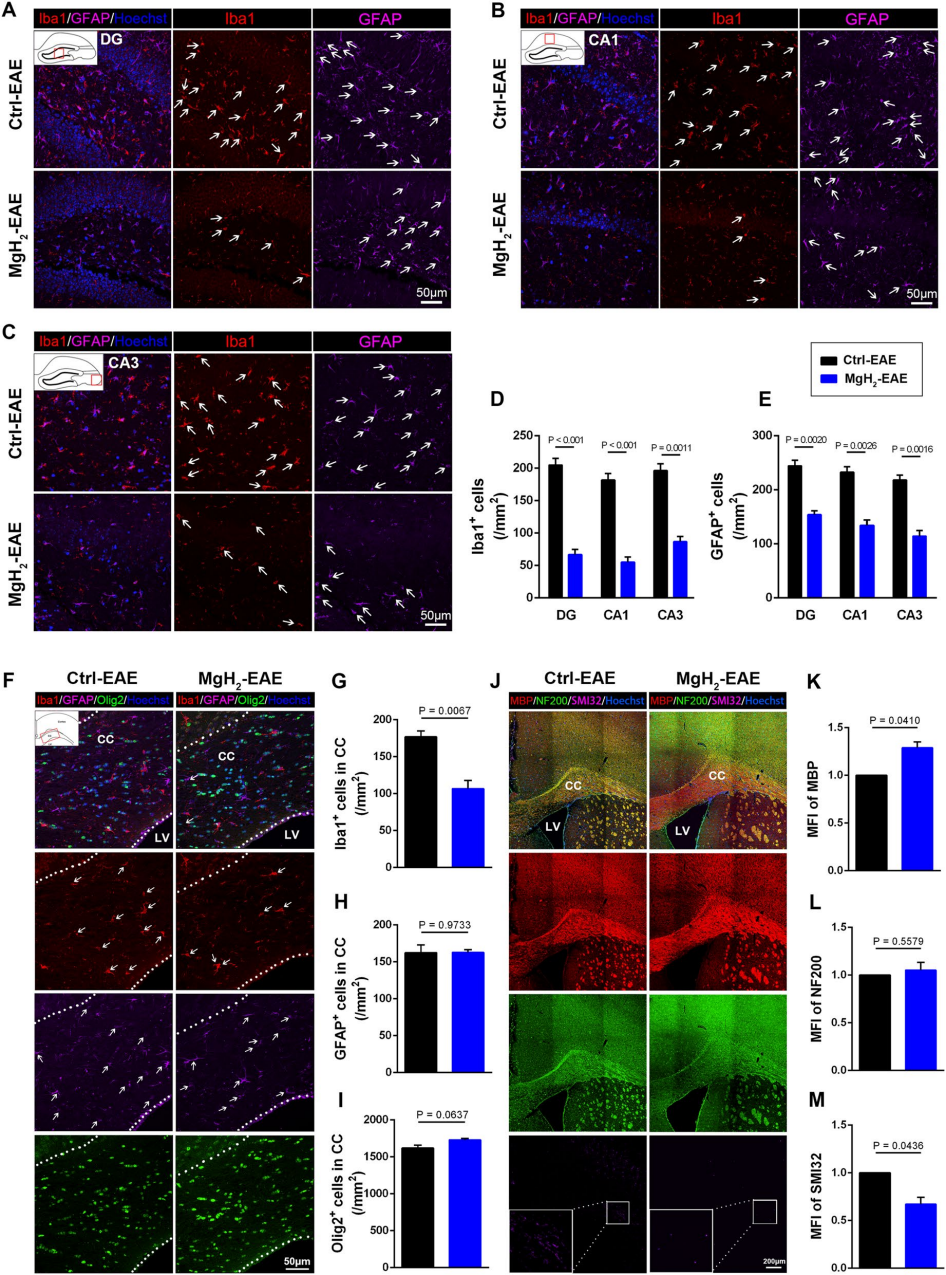
MgH_2_ treatment alleviates inflammation in the hippocampus and corpus callosum (CC) of EAE mice. **A–C** Representative anti-Iba1 (red) and anti-GFAP (purple) immunofluorescence of DG, CA1, and CA3 zones in the hippocampus. White arrows mark Iba1^+^ microglia and GFAP^+^ astrocytes. **D** Quantitative analysis of Iba1^+^ cells/mm^2^ of DG (*F* = 0.2353), CA1 (*F* = 0.1947) and CA3 (*F* = 0.1449) zone in the hippocampus, *n* = 3 mice per group. **E** Quantitative analysis of GFAP^+^ cells/mm^2^ of DG (*F* = 0.6708), CA1 (*F* = 0) and CA3 (*F* = 0.07) zone in the hippocampus, *n* = 3 mice per group. **F** Representative anti-Iba1 (red), anti-GFAP (purple), and anti-Olig2 (green) immunofluorescence of the CC, LV: lateral ventricle. **G–I** Quantitative analysis of Iba1^+^ cells/mm^2^ (*F* = 0.6852), GFAP^+^ cells/mm^2^ (*F* = 3.2429), and Olig2^+^ cells/mm^2^ (*F* = 0.81) of the CC, *n* = 3 mice per group. **J** Representative anti-MBP (red), anti-NF200 (green), and anti-SMI32 (purple) immunofluorescence of the CC. The difference of SMI32^+^ area is shown in white boxes. **K**–**M** Quantitative analysis of median fluorescence intensity (MFI) of MBP (*F* = 14.0746), NF200 (*F* = 4.5282), and SMI32 (*F* = 15.8079), *n* = 3 mice per group. White arrows mark Iba1^+^ or GFAP^+^ cells. Data are presented as mean ± SEM

Increasing research have shown that the hippocampus plays an important role in depression and anxiety [47–49]. Thus, mice were sacrificed at 11 dpi: after the behavioral assessments of anxiety and depression were completed and motor impairments began to appear. To evaluate the effect of MgH_2_ on neuroinflammation, we used Iba1 to mark microglia and GFAP to mark astrocytes in the hippocampus, including CA1, CA3, and DG. We found that the number of Iba1^+^ and GFAP^+^ cells in the MgH_2_-EAE group was significantly less than in the control-EAE group (Fig. 3A–E), showing that MgH_2_ treatment significantly reduced the inflammation in EAE mice.

One of the most replicated neurobiological findings in anxiety and depression is the structural abnormalities of the corpus callosum [50]. The corpus callosum plays a vital role in maintaining stable, functional communication between hemispheres [51]. We found that the number of Iba1^+^ cells in the corpus callosum of control-EAE mice was significantly greater than that of MgH_2_-EAE mice (Fig. 3F, G), but there was no difference in the density of astrocytes between the two groups (Fig. 3F, H). These data indicate that MgH_2_ treatment alleviates microglial activation in the corpus callosum of EAE mice.

Demyelination and neurodegeneration may also contribute to the progression of anxiety and depression in EAE. To evaluate the effect of MgH_2_ on white matter integrity after EAE, we examined the conditions of myelination and oligodendrocytes. Compared with the control-EAE mice, MgH_2_-EAE mice showed higher mean fluorescence intensity (MFI) of MBP (Fig. 3J, K). Although there was no statistically significant difference between the two groups, the number of Olig2^+^ cells in the MgH_2_-EAE group was slightly higher (Fig. 3F, I). Moreover, we used PDGF Receptor α (PDGFRα) to mark the oligodendrocyte progenitor cells (OPCs) and Ki67 to mark the proliferating cells. We found that there was no significant difference in PDGFRα^+^Sox10^+^ cells and Ki67^+^PDGFRα^+^Sox10^+^ cells between the control-EAE group and MgH_2_-EAE group (Additional file 1: Fig. S2A, B). Interestingly, the number of CC1^+^Sox10^+^ cells (mature oligodendrocytes) in the corpus callosum was increased in the MgH_2_-EAE group compared with the control-EAE group (Additional file 1: Fig. S2C, D), suggesting that the increased Olig2^+^ cells in the MgH_2_-EAE group were more likely to be mature oligodendrocytes. These results indicate that MgH_2_ treatment promotes maturation and myelination but did not affect the proliferation of OPCs after EAE.

At the same time, the MFI of Neurofilament 200 (NF200) did not differ between the two groups, but the MFI of non-phosphorylated neurofilament-H (SMI32), a marker of damaged axons, was significantly lower in the MgH_2_-EAE mice (Fig. 3J, L, M) suggesting less axonal degeneration in MgH_2_-EAE mice.

In summary, these results suggest that inflammation, myelin sheath loss, and damaged axons are potentially the mechanism for behavioral changes in EAE mice and that MgH_2_ treatment can reduce these pathological changes.

### MgH_2_ treatment improves depression-like behaviors induced by acute restraint

**Fig. 4:**
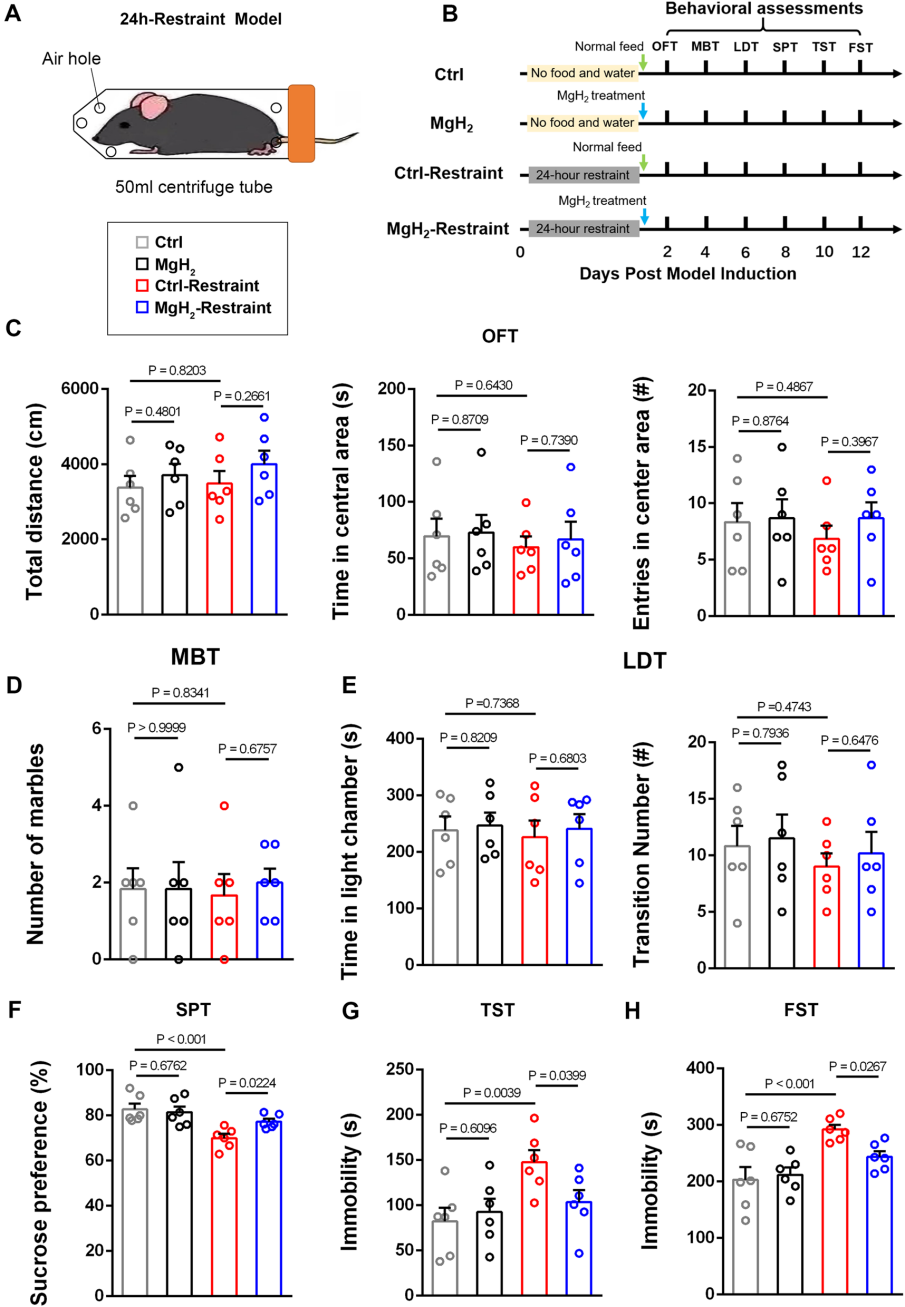
MgH_2_ treatment improves depressive-like behaviors induced by 24-h restraint. **A** Model of 24-h-restraint stress. **B** Schematic diagram displaying the behavioral testing in 4 different groups to test the effect of MgH2 treatment. **C** OFT, statistical analysis of total distance (*F* = 0.7394), time (*F* = 0.1454) and entries (*F* = 0.342) in the central area, *n* = 6 mice per group. **D** MBT, statistical analysis of the number of buried marbles (*F* = 0.0601), *n* = 6 mice per group. **E** Light/dark transition test (LDT), statistical analysis of time in the light chamber (*F* = 0.1163) and transition numbers (*F* = 0.3597), *n* = 6 mice per group. **F** SPT, statistical analysis of the percentage of sucrose consumption (*F* = 7.616), *n* = 6 mice per group. **G** TST, statistical analysis of immobile time (*F* = 4.1247), *n* = 6 mice per group. **H** Forced swimming test (FST), statistical analysis of immobile time (*F* = 7.9412), *n* = 6 mice per group. Data are presented as mean ± SEM

Considering that the antidepressant effect of MgH_2_ after EAE may be due to demyelination and axon damage in the CNS, we wanted to further explore the role of MgH_2_ in an acute restraint stress-induced depression model. To establish the acute restraint stress model, mice were confined to 50 mL centrifuge tubes and fasted for 24 h (Fig. 4A). Unlike our observations after EAE, we observed no difference in the MFI of MBP, NF200, and SMI32 in the corpus callosum after restraint and MgH_2_ treatment (Additional file 1: Fig. S3A–C). Meanwhile, there were no differences in the number of PDGFRα^+^Sox10^+^ cells, Ki67^+^PDGFRα^+^Sox10^+^ cells, and CC1^+^Sox10^+^ cells among three different groups (Additional file 1: Fig. S3D–G). To further explore the effect of MgH_2_ on the long-term proliferation of OPCs, we administered BrdU intraperitoneally to mice (Additional file 1: Fig. S4A). We found that neither restraint nor MgH_2_ treatment had any effect on the proliferation of OPCs (Additional file 1: Fig. S4B, C). These results suggest that acute restraint stress does not elicit demyelination or axon damage.

Behavioral tests were performed 2 d after restraint (Fig. 4B). The behavioral results of the OFT, MBT, and LDT showed that short-term restraint did not cause anxiety-like behaviors in the control-restraint group (Fig. 4C–E) compared with the control group. However, the control-restraint group distinctly preferred sucrose less than the control group (Fig. 4F). In the TST and forced swimming test (FST), measures of behavioral despair, the control-restraint group spent more time immobile (Fig. 4G, H). These findings indicate that the acute restraint stress model can induce depression-like behaviors. Significantly, the MgH_2_-restraint group performed better than the control-restraint group in the SPT, TST, and FST (Fig. 4F–H). In addition, we found that the MgH_2_ group (without restraint) showed similar behaviors to the control group (Fig. 4C–H). In summary, using several different behavioral tests, we found that MgH_2_ treatment did not affect mental status of normal mice but could improve depression-like symptoms after acute restraint.

### MgH_2_ treatment reduces inflammation in the CNS after acute restraint

**Fig. 5:**
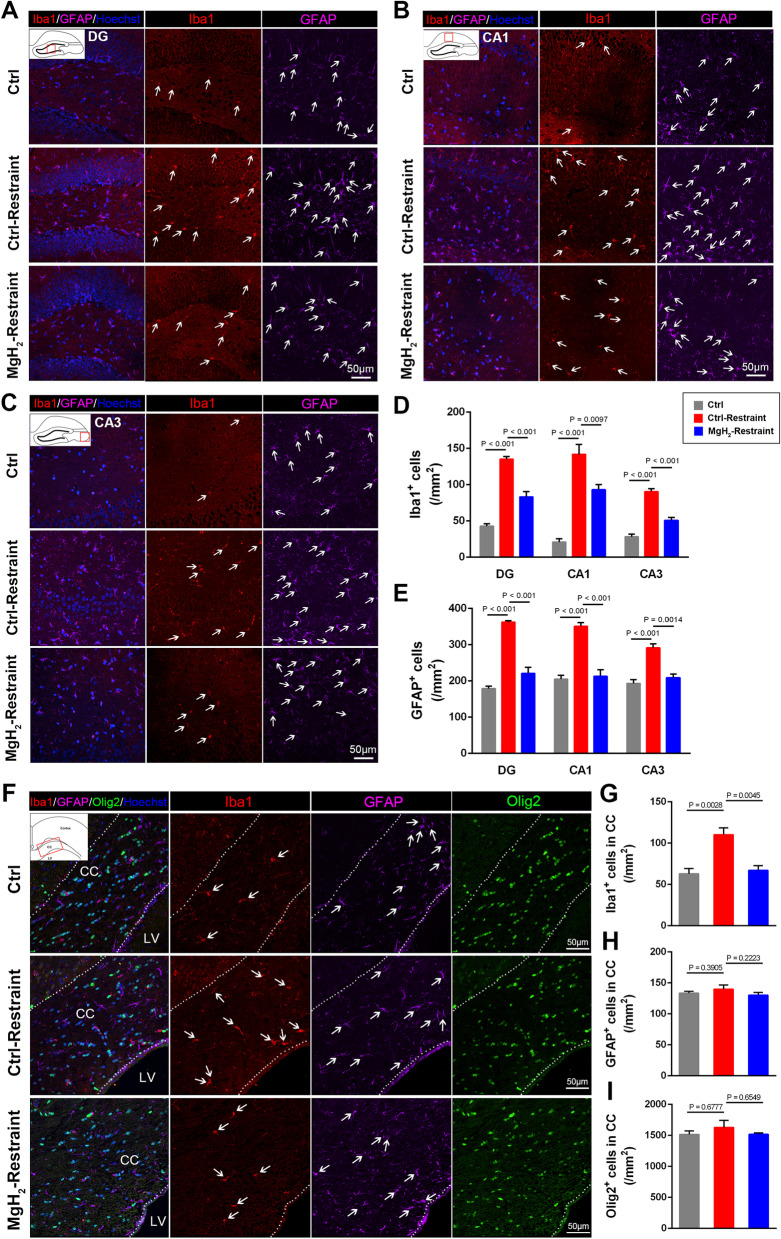
MgH_2_ treatment reduces inflammation of the hippocampus and corpus callosum after 24-h restraint. **A–C** Representative anti-Iba1 (red), anti-GFAP (purple) immunofluorescence of DG, CA1, and CA3 zones in the hippocampus. White arrows mark Iba1^+^ microglia and GFAP^+^ astrocytes. **D** Quantitative analysis of Iba1^+^ cells/mm^2^ of DG (*F* = 81.5234), CA1 (*F* = 43.3062) and CA3 (*F* = 64.6975) zones in the hippocampus, *n* = 3 mice per group. **E** Quantitative analysis of GFAP^+^ cells/mm^2^ of DG (*F* = 77.5573), CA1 (*F* = 37.1143) and CA3 (*F* = 25.7619) zones in the hippocampus, *n* = 3 mice per group. **F** Representative anti-Iba1 (red), anti-GFAP (purple), and anti-Olig2 (green) immunofluorescence of the CC. White arrows mark Iba1^+^ microglia and GFAP^+^ astrocytes. **G–I** Quantitative analysis of Iba1^+^ cells/mm^2^ (*F* = 14.4151), GFAP^+^ cells/mm^2^ (*F* = 0.9667) and Olig2^+^ cells/mm^2^ (*F* = 0.7709) of the CC, *n* = 3 vs. 3 mice per group. White arrows mark Iba1^+^ or GFAP^+^ cells. Data are presented as mean ± SEM

Stress is the primary environmental risk factor in the etiology of depression. Stress may also cause neuroinflammation, mainly by activating microglia [52]. After the 24 h restraint stress, we found that significantly more inflammatory cells infiltrated the hippocampus of the control-restraint group compared to that of the MgH_2_-restraint group (Additional file 1: Fig. S5A, B). Immunofluorescence results showed that the control-restraint group had significantly more Iba1^+^ microglia and GFAP^+^ astrocytes in the CA1, CA3, and DG regions of the hippocampus than the control group (Fig. 5A–E). Interestingly, these inflammatory changes in the hippocampus were less pronounced in the MgH_2_-restraint group (Fig. 5D, E). Moreover, more cells infiltrated the corpus callosum of the control-restraint group compared to that of the MgH_2_-restraint group (Additional file 1: Fig. S5C, D). As observed in the hippocampus, the number of microglia in the corpus callosum was significantly higher in the control-restraint group than in the control group (Fig. 5F, G). However, the number of astrocytes and oligodendrocytes did not change (Fig. 5F, H, I). After MgH_2_ treatment, the number of microglia observably decreased in the corpus callosum (Fig. 5F, G), but the number of astrocytes and oligodendrocytes were not affected (Fig. 5F, H, I). Together, these results suggest that MgH_2_ treatment can reduce the inflammation in the hippocampus and corpus callosum induced by acute restraint stress.

### MgH_2_ treatment does not affect the remyelination in the LPC model

**Fig. 6:**
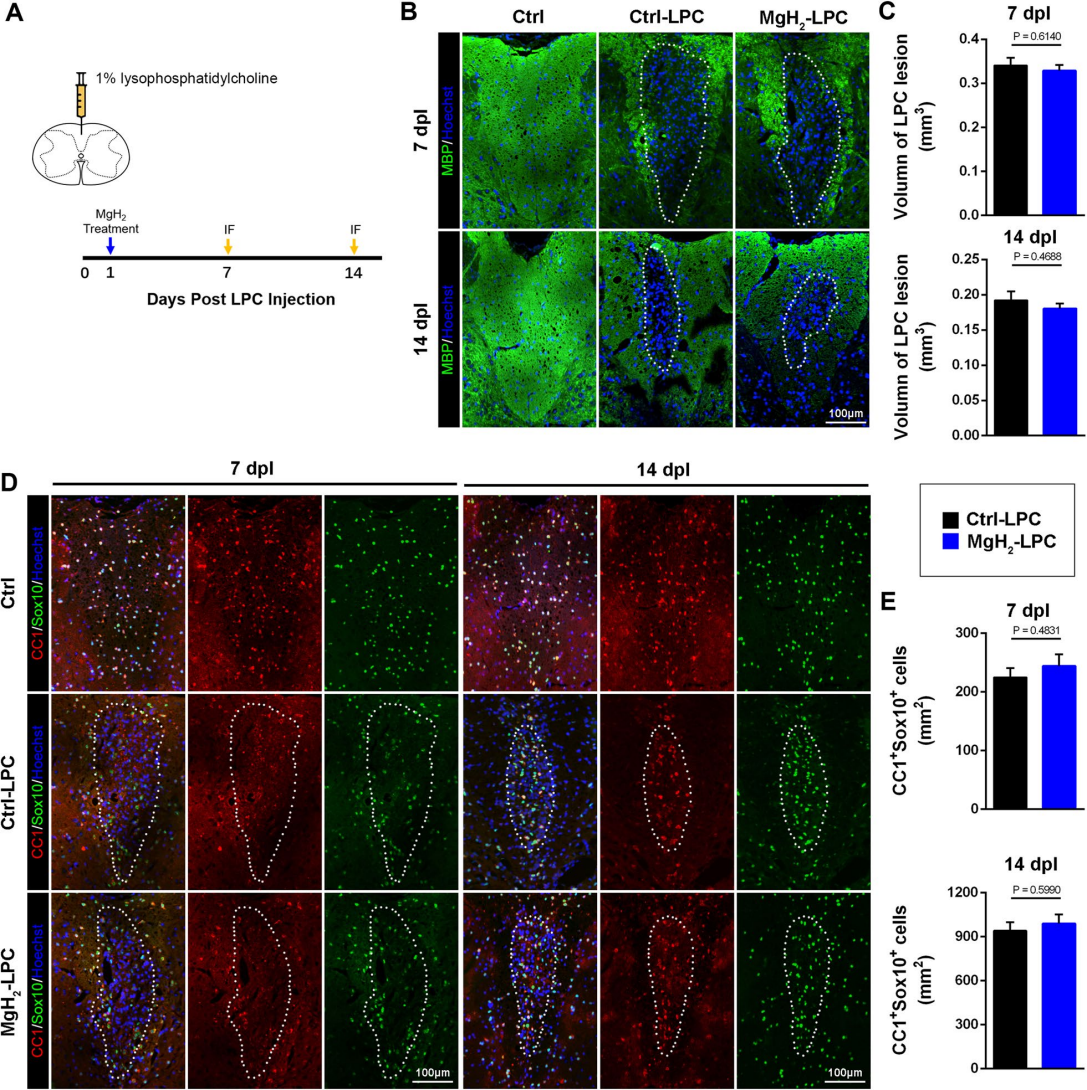
MgH_2_ treatment does not affect the remyelination in LPC-induced focal demyelination lesions. **A** Schematic diagram displaying the injection site and the timing of the LPC model. **B** Representative anti-MBP (green) immunofluorescence of the demyelinating region in the dorsal column of the spinal cord. **C** Quantitative analysis of the volume of demyelination at 7 dpl (*F* = 0.1788) and 14 dpl (*F* = 1.505), *n* = 3 mice per group. **D** Representative anti-CC1 (red), anti-Sox10 (green) immunofluorescence of the demyelinated region in the dorsal column of the spinal cord. **E** Quantitative analysis of CC1^+^Sox10^+^ cells/mm^2^ in the demyelinating lesions at 7 dpl (*F* = 0.2777) and 14 dpl (*F* = 0.0162), *n* = 3 mice per group. Data are presented as mean ± SEM

To further examine whether MgH_2_ plays a specific role in the myelination in vivo, we induced the LPC model (Fig. 6A). The LPC model displays the demyelination at 5–7-day post-lesion (dpl) and exhibits remyelination at 14 dpl [53]. We used MBP staining to assess white matter integrity and found that there was no significant difference in the demyelination volume between the ctrl-LPC group and the MgH_2_-LPC group at 7 dpl and 14 dpl (Fig. 6B, C). Likewise, no significant difference was observed in the number of CC1^+^Sox10^+^ cells in the lesions between the ctrl-LPC group and MgH_2_-LPC group at 7dpl and 14dpl following LPC (Fig. 6D, E). In summary, these results suggest that MgH_2_ treatment does not affect demyelination and remyelination after LPC modeling.

### MgH_2_ treatment affects microglial polarization and suppresses oxidative stress in vitro

**Fig. 7:**
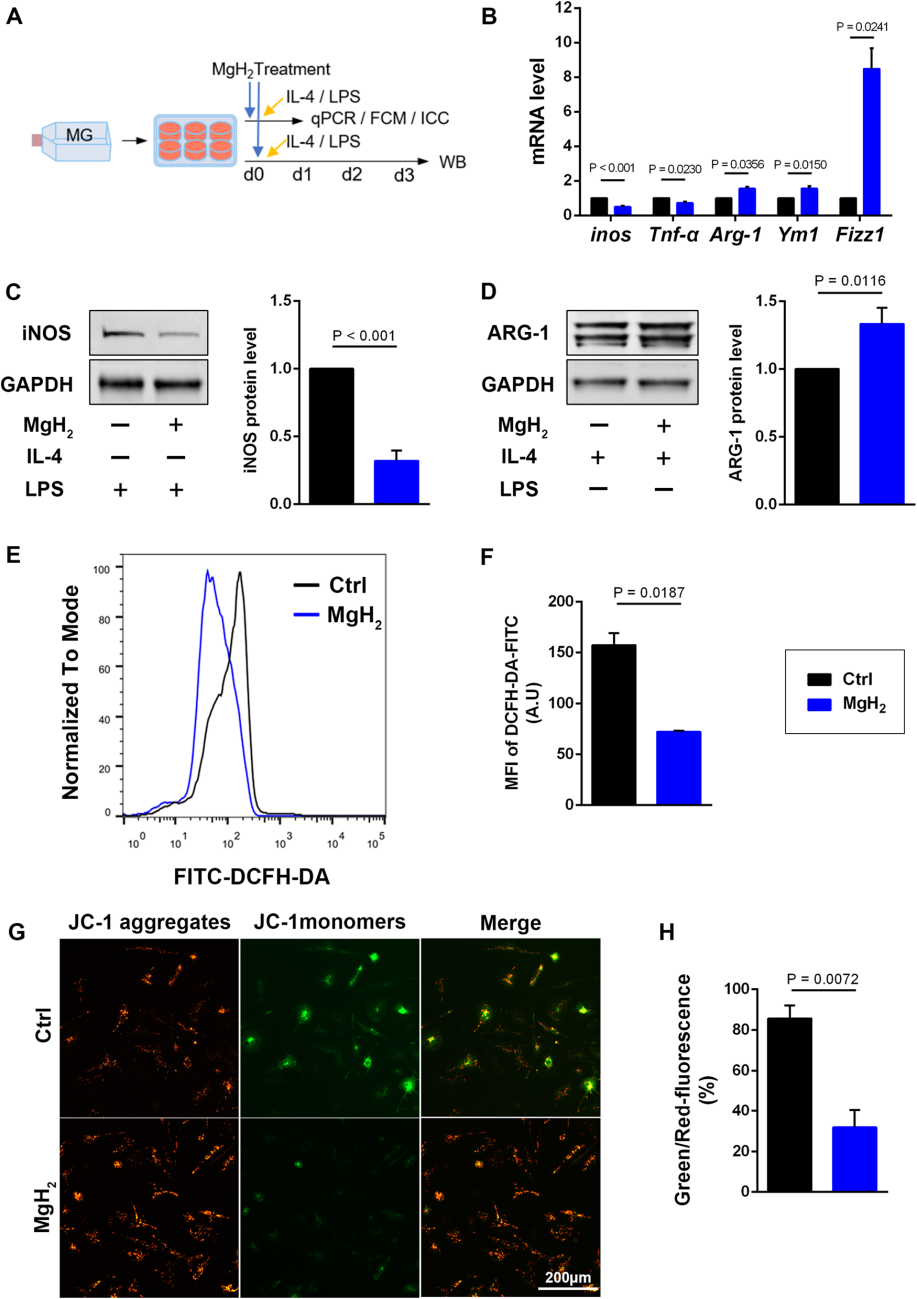
MgH_2_ treatment can affect polarization and inhibit oxidative stress of microglia. **A** Schematic diagram displaying the study on the polarization of microglia in vitro. **B** Relative *inos* (*F* = 4.3253), *Tnf-α* (*F* = 4.7244), *Arg-1* (*F* = 15.4525), *Ym1* (*F* = 6.8116), and *Fizz1* (*F* = 9.5084) mRNA expression in microglia in vitro at 24 h after IL-4 or LPS stimulation. *inos* and *Tnf-α* are used as M1 markers; *Arg-1*, *Ym1*, and *Fizz1* are used as M2 markers. *n* = 3 experiments per group. **C** Western blotting analysis and relative iNOS levels of microglia in vitro at 72 h (*F* = 5.3167), *n* = 3 experiments per group. **D** Western blotting analysis and relative ARG-1 protein levels of microglia in vitro at 72 h (*F* = 6.0097), *n* = 3 experiments per group. **E, F** Intracellular ROS of microglia is measured by flow cytometry (DCFH-DA fluorescent probe) and quantitative analysis of the MFI (*F* = 10.4918). *n* = 3 experiments per group. **G, H** Representative graphs show JC-1 fluorescence of microglia in vitro and quantitative analysis of green/red fluorescence intensity (%), red fluorescence represents the mitochondrial aggregate JC-1 and green fluorescence indicates the monomeric JC-1 (*F* = 0.2891). *n* = 3 experiments per group. Data are presented as mean ± SEM

To further explore the roles of MgH_2_ on microglial functions, we designed in vitro microglial polarization experiments (Fig. 7A). MgH_2_ was added to the culture medium of the MgH_2_ treatment group 1 h before LPS or IL-4 stimulation. QPCR analysis and western blotting analysis were performed 24 h and 72 h, respectively, after stimulation to microglial. QPCR analysis showed that the MgH_2_-treated microglia had lower expression of the M2 markers *Arginase-1*(*Arg-1*), *chitinase 3-like-3* (*Ym1*)*,* and *found in inflammatory zone 1* (*Fizz1*), and higher expression of the M1 markers *inducible nitric oxide synthase* (*inos*) and *transforming growth factor-α* (*Tnf-α*) compared to the control group (Fig. 7B). Western blotting showed similar changes in iNOS and ARG-1 protein expression (Fig. 7C, D). These molecular results suggest that MgH_2_ suppresses microglial M1 polarization and promotes microglial M2 polarization.

Then, we detected microglial intracellular ROS levels using DCFH-DA by flow cytometry analysis after LPS stimulation. As shown in Fig. 7E, F, ROS generation was lower in the MgH_2_-treated microglia than compared with the control group. Depolarization of mitochondrial membrane potential (MMP) is a hallmark of mitochondrial dysfunction. When MMP is depolarized, accumulation of JC-1 in mitochondria is reduced, representing decreased red fluorescent aggregates, and increased green fluorescent monomers [54]. While the MgH_2_-treated microglia showed little green fluorescence indicating relative normal MMP after LPS stimulation, the controls emitted strong green fluorescence indicating depolarization (Fig. 7G, H).

Together, these results indicate that MgH_2_ treatment can affect microglial polarization, suppress microglial oxidative stress, and protect mitochondrial function, partially explaining how MgH_2_ inhibits inflammation in vivo.

### MgH_2_ does not affect the behaviours of OPCs in vitro

We observed that there was a decrease in myelin content and the number of mature oligodendrocytes after EAE, and these changes were partially reversed by MgH_2_ treatment according to in vivo experiments mentioned above. However, MgH_2_ did not appear to affect remyelination in the LPC model. Since the cellular source of remyelination in CNS is OPCs, we then assessed the direct effects of MgH_2_ on OPCs in vitro (Additional file 1: Fig. S6A).

To investigate whether MgH_2_ regulates the proliferation and apoptosis of OPCs, BrdU incorporation assay and Caspase3 immunofluorescence staining were performed. No difference in the ratio of BrdU^+^ cells and Caspase3^+^ cells between the MgH_2_-treated OPCs and the control cells was detected (Additional file 1: Fig. S6B–D). As the major cellular function of OPCs is to differentiate into myelinated oligodendrocytes, we also tested the effect of MgH_2_ on OPCs differentiation. Immunofluorescence staining showed no difference in MBP^+^ mature oligodendrocytes between the MgH_2_-treated and control groups, which were significantly lower than those in the T3 group (Additional file 1: Fig. S6E). Western blotting analysis also confirmed that myelin-associated glycoprotein (MAG) and MBP protein expression levels were comparable in the MgH_2_-treated and control groups (Additional file 1: Fig. S6F). In summary, these in vitro results are consistent with the results from the LPC model, indicating that MgH_2_ does not influence OPCs functions and may indirectly modulate the remyelination process.

## Discussion

Mental symptoms such as anxiety and depression are part of MS symptomatology and can also occur as comorbidities. Mental comorbidities of MS demand urgent attention, since they contribute significantly to secondary disability and the decline of life quality [5, 55, 56]. Neuroinflammation, especially microglia activation and dysfunction in mood-related brain regions of MS patients may account for the development of depressive and anxious symptoms [57–59]. In the present study, we found that MgH_2_ could significantly reduce CNS inflammation, alleviate anxiety/depression-like symptoms and relieve the progress of EAE by modulating microglial polarization, suppressing oxidative stress, and reducing mitochondrial damage.

As a broadly adopted animal model of MS, EAE also mimic the mood disorders in MS patients which can be observed in EAE mice before 10 dpi (the mice develop motor dysfunction after 10 dpi). During this period, the mice gradually developed anxiety/depression-like psychiatric symptoms. It was reported that EAE mice with mental impairment have neuronal loss in the hippocampus, microglial activation in the striatum, elevated levels of IL-1β in the hypothalamus, and increased TNF-α in these brain regions [60–62]. Our results showed that anxiety/depression-like psychiatric symptoms in EAE mice are accompanied with extensive neuroinflammation in the hippocampus and corpus callosum, including activation of microglia and astrocytes and mild damage of myelin and axon integrity. These findings further suggest that neuroinflammation may play a significant role in the development of mental disorders in MS patients. Notably, we found that EAE induction reduced the movement of mice during the pre-onset phase. Considering the normal clinical EAE scores during the pre-onset phase in Ctrl-EAE mice, we believe that the movement reduction is likely to be one of the anxiety/depression-like psychiatric symptoms, but not a motor dysfunction. In addition, our results support the notion that depressive symptoms are not merely the reactive epiphenomenon of MS pathology, since the depressive symptoms emerged even before the onset of MS.

Our study demonstrated that MgH_2_ could alleviate motor dysfunction, inflammatory demyelination, and emotional symptoms in EAE mice. However, it is unclear whether the antidepressant and anxiolytic effect of MgH_2_ is due to reduced demyelinating damage or an anti-inflammatory effect, or both. We established acute restraint stress model and LPC model to answer this question. Our study validated that depression-like symptoms could be induced by the acute restraint stress model. Moreover, we found no significant structural abnormalities, such as demyelination and axonal injury, in the corpus callosum of mice after acute restraint. Activation of microglia and astrocytes in the CNS of restrained mice suggests that inflammation is closely associated with depression. Moreover, the results from LPC experiments indicated that MgH_2_ did not affect oligodendrocyte differentiation and CNS remyelination, which could also be inferred from in vitro OPCs experiment. However, reduced demyelination, increased mature oligodendrocytes, and enhancement of myelin-associated proteins in both the spinal cord and corpus callosum were observed in EAE mice treated with MgH_2_. There are two possible explanations: first, MgH_2_ administered at the beginning of EAE induction may reduce the occurrence of inflammatory demyelination; second, anti-inflammatory treatment of MgH_2_ may provide a favorable microenvironment for remyelination. Our data verified that MgH_2_ was most likely to suppress anxiety/depression-like symptoms of EAE through modulating the inflammatory response rather than remyelination. Besides, these results, following the previous in-depth research on the neurobiological mechanism of depression, show that neuroinflammation plays an important role in the occurrence and development of depression [63–66].

Although various types of cells respond to neuroinflammation, microglia are activated earlier than other glial cells under noxious stimuli or pathological conditions [67, 68]. Some scholars believe that neuroinflammation in the brain is triggered and maintained by microglia activation. Furthermore, microglial M1 and M2 polarization is also an important component of neuroinflammation [69, 70], whereas astrocytes appear to be more tolerant than microglia in response to inflammatory stimuli [71]. Consistent with the literature, the neuroinflammation observed in the EAE and restraint stress models is composed of extensive microglial activation and moderate astrocyte activation. Our results show that microglia and astrocytes are activated to different degrees in both EAE and restraint stress models in the hippocampus. However, the microglia and astrocytes in the corpus callosum seem to be less responsive. Thus, our findings strongly suggest that the hippocampus is more vulnerable to acute and chronic inflammation than the corpus callosum, even though both regions are critical in mental disorders.

Magnesium plays a significant role in regulating the function of immune cells [72, 73]. Previous studies have shown that magnesium can inhibit proinflammatory factors and promote M2 polarization in the brain [74–77]. Multiple studies have also confirmed the anti-inflammatory effects of H_2_ in the CNS [29]. However, the effects of H_2_ on microglial polarization are controversial, perhaps due to different diseases or animal models. In a stroke model, H_2_ significantly inhibited M1 polarization but had a lesser effect on M2 polarization [78]. Another study noted that hydrogen-rich saline might promote microglial M2 polarization in newborn mice exposed to hypoxic-ischemic conditions [79]. Our study first explored the effect of MgH_2_ on microglial polarization. The results show that MgH_2_ can inhibit microglia M1 polarization and promote M2 polarization in vitro, which may be due to the synergistic effect of H_2_ and magnesium. Neuroinflammation is usually accompanied with oxidative stress. Oxidative stress results from an imbalance between the production and clearance of ROS which is mainly from mitochondrial oxidative metabolism. Our study indicates that MgH_2_ can reduce the level of microglial ROS and mitochondrial damage in vitro. However, the specific mechanism of MgH_2_’s anti-inflammatory and antioxidant effects remains to be further investigated.

## Conclusions

Our study shows that nanoparticulate MgH_2_ can modulate microglial polarization, decrease oxidative stress, and effectively alleviate the anxiety/depression-like symptoms of EAE mice through anti-inflammatory effects. Thus, nanoparticulate MgH_2_ might represent a safe and translational amenable therapeutic option for MS comorbid with anxiety and depression.

## Supplementary Information

**Additional file 1: Figure S1.** Confirming the concentration of MgH_2_ in vitro. Cell viability of BV2 in different concentrations of MgH_2_ (*F* = 16.26), *n* = at least 5 repeats per group. Data are presented as mean ± SEM. **Figure S2.** MgH_2_ treatment promotes the maturation of OPCs in EAE mice. (A) Representative anti-Ki67 (gray), anti-PDGFRα (red), and anti-Sox10 (green) immunofluorescence of the CC. (B) Quantitative analysis of PDGFRα^+^Sox10^+^ cells/mm^2^ (*F* = 0.4957) and Ki67^+^PDGFRα^+^Sox10^+^ cells/mm^2^ (*F* = 0.4059), *n* = 3 mice per group. (C) Representative anti-CC1 (red) and anti-Sox10 (green) immunofluorescence of the CC. (D) Quantitative analysis of CC1^+^Sox10^+^ cells/mm^2^ (*F* = 2.7514), *n* = 3 mice per group. Data are presented as mean ± SEM. **Figure S3.** Myelination and axons do not alter after 24-h restraint stress and MgH_2_ treatment. (A) Representative anti-MBP (red), anti-NF200 (green), and anti-SMI32 (purple) immunofluorescence of the CC. (B, C) Quantitative analysis of the MFI of MBP (*F* = 0.092) and NF200 (*F* = 0.0109), *n* = 3 mice per group. (D) Representative anti-Ki67 (gray), anti-PDGFRα (red), and anti-Sox10 (green) immunofluorescence of the CC. (E) Quantitative analysis of PDGFRα^+^Sox10^+^ cells/mm^2^ (*F* = 0.0323) and Ki67^+^PDGFRα^+^Sox10^+^ cells/mm^2^ (*F* = 0.1672), *n* = 3 mice per group. (F) Representative anti-CC1 (red) and anti-Sox10 (green) immunofluorescence of the CC. (G) Quantitative analysis of CC1^+^Sox10^+^ cells/mm^2^ (*F* = 0.1775), *n* = 3 mice per group. Data are presented as mean ± SEM. **Figure S4.** MgH_2_ treatment has no effect on the long-term proliferation of OPCs after 24-h restraint stress. (A) Schematic diagram displaying the cell proliferation test in 4 different groups. (B) Representative anti-BrdU (red), anti-Olig2 (green) and anti-Sox10 (purple) immunofluorescence of the CC. (C) Quantitative analysis of BrdU^+^Olig2^+^Sox10^+^ cells/mm^2^ (*F* = 0.1466), *n* = 3 mice per group. Data are presented as mean ± SEM. **Figure S5.** Number of infiltrating cells is reduced by MgH_2_ treatment after 24-h restraint stress. (A) Representative brain sections of H&E staining in different regions of the hippocampus. (B) Quantitative analysis of the number of infiltrating cells in the hippocampus (*F* = 0.3478), *n* = 3 mice per group. (C) Representative brain sections of H&E staining in the CC. (D) Quantitative analysis of the number of infiltrating cells in the CC (*F* = 2.1923), *n* = 3 vs. 3 mice per group. Data are presented as mean ± SEM. **Figure S6.** MgH_2_ treatment does not affect OPCs functions in vitro. (A) Schematic diagram displaying the study on functions of OPCs in vitro. (B) Representative anti-BrdU (red) and anti-Caspase3 (purple) immunofluorescence of OPCs in vitro at 48 h (in proliferation medium) and 72 h (in differentiation medium), respectively. (C, D) Quantitative analysis of the percentages of BrdU^+^ cells (*F* = 0.4577) and Caspase3^+^ cells (*F* = 2.2225) in (B), *n* = 1076 vs. 1014 cells (BrdU), *n* = 1145 vs. 1217 cells (Caspase3). (E) Representative anti-MBP (green) immunofluorescence of OPCs in vitro at 72 h (in differentiation medium). (F) Western blotting analysis relative MBP (*F* = 2.2225) and MAG (*F* = 47.8691) protein levels of OPCs in vitro at 72 h (in differentiation medium), *n* = 3 experiments per group. Data are presented as mean ± SEM.

## Data Availability

Supporting data and information about used material are available from the corresponding author on reasonable request.

## Abbreviations

MS: Multiple sclerosis
CNS: Central nervous system
EAE: Experimental autoimmune encephalomyelitis
LPC: Lysophosphatidylcholine
WM: White matter
H&E: Hematoxylin and eosin
LFB: Luxol fast blue
OFT: Open field test
MBT: Marble burying test
LDT: Light/dark transition test
SPT: Sucrose preference test
TST: Tail suspension test
FST: Forced swimming test
CC: Corpus callosum
CA: Cornus ammonis
DG: Dentate gyrus
MFI: Mean fluorescence intensity
Iba1: Ionized calcium-binding adaptor protein-1
GFAP: Glial fibrillary acidic protein
Sox10: SRY-Box Transcription Factor 10
CC1 (aka APC): Adenomatous polyposis coli
MBP: Myelin basic protein
MAG: Myelin associated glycoprotein
NF200: Neurofilament 200
ARG-1: Arginase-1
iNOS: Inducible nitric oxide synthase
Ym1: Chitinase 3-like-3
Fizz1: Found in inflammatory zone 1
Tnf-α: Transforming growth factor-α
IL-4: Interleukin-4
LPS: Lipopolysaccharide
ROS: Reactive oxygen species
MMP: Mitochondrial membrane potential
OPCs: Oligodendrocyte progenitor cells
DCFH-DA: 2,7-Dichlorodihydrofluorescein diacetate

## References

[1] Compston A, Coles A (2002). Multiple sclerosis. Lancet.

[2] Mohr DC, Hart SL, Fonareva I, Tasch ES (2006). Treatment of depression for patients with multiple sclerosis in neurology clinics. Mult Scler.

[3] Marrie RA, Reingold S, Cohen J, Stuve O, Trojano M, Sorensen PS, Cutter G, Reider N (2015). The incidence and prevalence of psychiatric disorders in multiple sclerosis: a systematic review. Mult Scler.

[4] Magyari M, Sorensen PS (2020). Comorbidity in multiple sclerosis. Front Neurol.

[5] Feinstein A, Magalhaes S, Richard JF, Audet B, Moore C (2014). The link between multiple sclerosis and depression. Nat Rev Neurol.

[6] Chou IJ, Kuo CF, Tanasescu R, Tench CR, Tiley CG, Constantinescu CS, Whitehouse WP (2020). Comorbidity in multiple sclerosis: its temporal relationships with disease onset and dose effect on mortality. Eur J Neurol.

[7] Kalb R, Feinstein A, Rohrig A, Sankary L, Willis A (2019). Depression and suicidality in multiple sclerosis: red flags, management strategies, and ethical considerations. Curr Neurol Neurosci Rep.

[8] Chwastiak L, Ehde DM, Gibbons LE, Sullivan M, Bowen JD, Kraft GH (2002). Depressive symptoms and severity of illness in multiple sclerosis: epidemiologic study of a large community sample. Am J Psychiatry.

[9] Coplan JD, Aaronson CJ, Panthangi V, Kim Y (2015). Treating comorbid anxiety and depression: psychosocial and pharmacological approaches. World J Psychiatry.

[10] Cipriani A, Furukawa TA, Salanti G, Chaimani A, Atkinson LZ, Ogawa Y, Leucht S, Ruhe HG, Turner EH, Higgins JPT, Egger M, Takeshima N, Hayasaka Y, Imai H, Shinohara K, Tajika A, Ioannidis JPA, Geddes JR (2018). Comparative efficacy and acceptability of 21 antidepressant drugs for the acute treatment of adults with major depressive disorder: a systematic review and network meta-analysis. Lancet.

[11] Zhou X, Teng T, Zhang Y, Del Giovane C, Furukawa TA, Weisz JR, Li X, Cuijpers P, Coghill D, Xiang Y, Hetrick SE, Leucht S, Qin M, Barth J, Ravindran AV, Yang L, Curry J, Fan L, Silva SG, Cipriani A, Xie P (2020). Comparative efficacy and acceptability of antidepressants, psychotherapies, and their combination for acute treatment of children and adolescents with depressive disorder: a systematic review and network meta-analysis. Lancet Psychiatry.

[12] Hetrick SE, McKenzie JE, Bailey AP, Sharma V, Moller CI, Badcock PB, Cox GR, Merry SN, Meader N (2021). New generation antidepressants for depression in children and adolescents: a network meta-analysis. Cochrane Database Syst Rev.

[13] Hashimoto K (2019). Rapid-acting antidepressant ketamine, its metabolites and other candidates: a historical overview and future perspective. Psychiatry Clin Neurosci.

[14] Nathoo N, Mackie A (2017). Treating depression in multiple sclerosis with antidepressants: a brief review of clinical trials and exploration of clinical symptoms to guide treatment decisions. Mult Scler Relat Disord.

[15] Fiest KM, Walker JR, Bernstein CN, Graff LA, Zarychanski R, Abou-Setta AM, Patten SB, Sareen J, Bolton JM, Marriott JJ, Fisk JD, Singer A, Marrie RA, Burden CTDT, D. Managing the Effects of Psychiatric Comorbidity in Chronic Immunoinflammatory (2016). Systematic review and meta-analysis of interventions for depression and anxiety in persons with multiple sclerosis. Mult Scler Relat Disord.

[16] Gosselin D, Skola D, Coufal NG, Holtman IR, Schlachetzki JCM, Sajti E, Jaeger BN, O'Connor C, Fitzpatrick C, Pasillas MP, Pena M, Adair A, Gonda DD, Levy ML, Ransohoff RM, Gage FH, Glass CK (2017). An environment-dependent transcriptional network specifies human microglia identity. Science.

[17] Singh S, Metz I, Amor S, van der Valk P, Stadelmann C, Bruck W (2013). Microglial nodules in early multiple sclerosis white matter are associated with degenerating axons. Acta Neuropathol.

[18] Voet S, Prinz M, van Loo G (2019). Microglia in central nervous system inflammation and multiple sclerosis pathology. Trends Mol Med.

[19] Xia Z, Friedlander RM (2017). Minocycline in multiple sclerosis—compelling results but too early to tell. N Engl J Med.

[20] Yirmiya R, Rimmerman N, Reshef R (2015). Depression as a microglial disease. Trends Neurosci.

[21] Felger JC (2018). Imaging the role of inflammation in mood and anxiety-related disorders. Curr Neuropharmacol.

[22] Muller N, Schwarz MJ (2007). The immune-mediated alteration of serotonin and glutamate: towards an integrated view of depression. Mol Psychiatry.

[23] Jia X, Gao Z, Hu H (2021). Microglia in depression: current perspectives. Sci China Life Sci.

[24] McKim DB, Weber MD, Niraula A, Sawicki CM, Liu X, Jarrett BL, Ramirez-Chan K, Wang Y, Roeth RM, Sucaldito AD, Sobol CG, Quan N, Sheridan JF, Godbout JP (2018). Microglial recruitment of IL-1beta-producing monocytes to brain endothelium causes stress-induced anxiety. Mol Psychiatry.

[25] Yasumoto Y, Stoiljkovic M, Kim JD, Sestan-Pesa M, Gao XB, Diano S, Horvath TL (2021). Ucp2-dependent microglia-neuronal coupling controls ventral hippocampal circuit function and anxiety-like behavior. Mol Psychiatry.

[26] Bai S, Guo W, Feng Y, Deng H, Li G, Nie H, Guo G, Yu H, Ma Y, Wang J, Chen S, Jing J, Yang J, Tang Y, Tang Z (2020). Efficacy and safety of anti-inflammatory agents for the treatment of major depressive disorder: a systematic review and meta-analysis of randomised controlled trials. J Neurol Neurosurg Psychiatry.

[27] Husain MI, Chaudhry IB, Husain N, Khoso AB, Rahman RR, Hamirani MM, Hodsoll J, Qurashi I, Deakin JF, Young AH (2017). Minocycline as an adjunct for treatment-resistant depressive symptoms: a pilot randomised placebo-controlled trial. J Psychopharmacol.

[28] Sorensen PS, Sellebjerg F, Lycke J, Farkkila M, Creange A, Lund CG, Schluep M, Frederiksen JL, Stenager E, Pfleger C, Garde E, Kinnunen E, Marhardt K, Investigators RS (2016). Minocycline added to subcutaneous interferon beta-1a in multiple sclerosis: randomized RECYCLINE study. Eur J Neurol.

[29] Chen W, Zhang HT, Qin SC (2021). Neuroprotective effects of molecular hydrogen: a critical review. Neurosci Bull.

[30] Zhang Y, Su WJ, Chen Y, Wu TY, Gong H, Shen XL, Wang YX, Sun XJ, Jiang CL (2016). Effects of hydrogen-rich water on depressive-like behavior in mice. Sci Rep.

[31] Pilchova I, Klacanova K, Tatarkova Z, Kaplan P, Racay P (2017). The involvement of Mg(2+) in regulation of cellular and mitochondrial functions. Oxid Med Cell Longev.

[32] Nielsen FH (2018). Magnesium deficiency and increased inflammation: current perspectives. J Inflamm Res.

[33] Shahi A, Aslani S, Ataollahi M, Mahmoudi M (2019). The role of magnesium in different inflammatory diseases. Inflammopharmacology.

[34] Zheltova AA, Kharitonova MV, Iezhitsa IN, Spasov AA (2016). Magnesium deficiency and oxidative stress: an update. Biomedicine (Taipei).

[35] Du J, Zhu M, Bao H, Li B, Dong Y, Xiao C, Zhang GY, Henter I, Rudorfer M, Vitiello B (2016). The role of nutrients in protecting mitochondrial function and neurotransmitter signaling: implications for the treatment of depression, PTSD, and suicidal behaviors. Crit Rev Food Sci Nutr.

[36] Eby GA, Eby KL (2010). Magnesium for treatment-resistant depression: a review and hypothesis. Med Hypotheses.

[37] Yu Z, Sun D, Feng J, Tan W, Fang X, Zhao M, Zhao X, Pu Y, Huang A, Xiang Z, Cao L, He C (2015). MSX3 switches microglia polarization and protects from inflammation-induced demyelination. J Neurosci.

[38] Chu X, Zhou Y, Hu Z, Lou J, Song W, Li J, Liang X, Chen C, Wang S, Yang B, Chen L, Zhang X, Song J, Dong Y, Chen S, He L, Xie Q, Chen X, Li W (2016). 24-hour-restraint stress induces long-term depressive-like phenotypes in mice. Sci Rep.

[39] Lu F, Yin D, Pu Y, Liu W, Li Z, Shao Q, He C, Cao L (2019). Shikimic acid promotes oligodendrocyte precursor cell differentiation and accelerates remyelination in mice. Neurosci Bull.

[40] Wiebe S, Nagpal A, Truong VT, Park J, Skalecka A, He AJ, Gamache K, Khoutorsky A, Gantois I, Sonenberg N (2019). Inhibitory interneurons mediate autism-associated behaviors via 4E-BP2. Proc Natl Acad Sci U S A.

[41] Crawley JN (1981). Neuropharmacologic specificity of a simple animal model for the behavioral actions of benzodiazepines. Pharmacol Biochem Behav.

[42] Fan KQ, Li YY, Wang HL, Mao XT, Guo JX, Wang F, Huang LJ, Li YN, Ma XY, Gao ZJ, Chen W, Qian DD, Xue WJ, Cao Q, Zhang L, Shen L, Zhang L, Tong C, Zhong JY, Lu W, Lu L, Ren KM, Zhong G, Wang Y, Tang M, Feng XH, Chai RJ, Jin J (2019). Stress-induced metabolic disorder in peripheral CD4(+) T cells leads to anxiety-like behavior. Cell.

[43] Jamain S, Radyushkin K, Hammerschmidt K, Granon S, Boretius S, Varoqueaux F, Ramanantsoa N, Gallego J, Ronnenberg A, Winter D, Frahm J, Fischer J, Bourgeron T, Ehrenreich H, Brose N (2008). Reduced social interaction and ultrasonic communication in a mouse model of monogenic heritable autism. Proc Natl Acad Sci U S A.

[44] Aguilar-Valles A, De Gregorio D, Matta-Camacho E, Eslamizade MJ, Khlaifia A, Skaleka A, Lopez-Canul M, Torres-Berrio A, Bermudez S, Rurak GM, Simard S, Salmaso N, Gobbi G, Lacaille JC, Sonenberg N (2021). Antidepressant actions of ketamine engage cell-specific translation via eIF4E. Nature.

[45] Sun D, Yu Z, Fang X, Liu M, Pu Y, Shao Q, Wang D, Zhao X, Huang A, Xiang Z, Zhao C, Franklin RJ, Cao L, He C (2017). LncRNA GAS5 inhibits microglial M2 polarization and exacerbates demyelination. EMBO Rep.

[46] Piras G, Rattazzi L, McDermott A, Deacon R, D'Acquisto F (2013). Emotional change-associated T cell mobilization at the early stage of a mouse model of multiple sclerosis. Front Immunol.

[47] Troubat R, Barone P, Leman S, Desmidt T, Cressant A, Atanasova B, Brizard B, El Hage W, Surget A, Belzung C, Camus V (2021). Neuroinflammation and depression: a review. Eur J Neurosci.

[48] Zhao X, Cao F, Liu Q, Li X, Xu G, Liu G, Zhang Y, Yang X, Yi S, Xu F, Fan K, Ma J (2019). Behavioral, inflammatory and neurochemical disturbances in LPS and UCMS-induced mouse models of depression. Behav Brain Res.

[49] Li M, Li C, Yu H, Cai X, Shen X, Sun X, Wang J, Zhang Y, Wang C (2017). Lentivirus-mediated interleukin-1beta (IL-1beta) knock-down in the hippocampus alleviates lipopolysaccharide (LPS)-induced memory deficits and anxiety- and depression-like behaviors in mice. J Neuroinflammation.

[50] Zhao W, Zhu D, Zhang Y, Zhang C, Zhang B, Yang Y, Zhu J, Yu Y (2021). Relationship between illness duration, corpus callosum changes, and sustained attention dysfunction in major depressive disorder. Quant Imaging Med Surg.

[51] Shen K, Misic B, Cipollini BN, Bezgin G, Buschkuehl M, Hutchison RM, Jaeggi SM, Kross E, Peltier SJ, Everling S, Jonides J, McIntosh AR, Berman MG (2015). Stable long-range interhemispheric coordination is supported by direct anatomical projections. Proc Natl Acad Sci U S A.

[52] Nieto-Quero A, Chaves-Pena P, Santin LJ, Perez-Martin M, Pedraza C (2021). Do changes in microglial status underlie neurogenesis impairments and depressive-like behaviours induced by psychological stress? A systematic review in animal models. Neurobiol Stress.

[53] Zhao C, Ma D, Zawadzka M, Fancy SP, Elis-Williams L, Bouvier G, Stockley JH, de Castro GM, Wang B, Jacobs S, Casaccia P, Franklin RJ (2015). Sox2 sustains recruitment of oligodendrocyte progenitor cells following CNS demyelination and primes them for differentiation during remyelination. J Neurosci.

[54] Liu S, Liu S, He B, Li L, Li L, Wang J, Cai T, Chen S, Jiang H (2021). OXPHOS deficiency activates global adaptation pathways to maintain mitochondrial membrane potential. EMBO Rep.

[55] Salehpoor G, Rezaei S, Hosseininezhad M (2014). Quality of life in multiple sclerosis (MS) and role of fatigue, depression, anxiety, and stress: a bicenter study from north of Iran. Iran J Nurs Midwifery Res.

[56] Solaro C, Gamberini G, Masuccio FG (2018). Depression in multiple sclerosis: epidemiology, aetiology, diagnosis and treatment. CNS Drugs.

[57] Vattakatuchery JJ, Rickards H, Cavanna AE (2011). Pathogenic mechanisms of depression in multiple sclerosis. J Neuropsychiatry Clin Neurosci.

[58] Colasanti A, Guo Q, Giannetti P, Wall MB, Newbould RD, Bishop C, Onega M, Nicholas R, Ciccarelli O, Muraro PA, Malik O, Owen DR, Young AH, Gunn RN, Piccini P, Matthews PM, Rabiner EA (2016). Hippocampal neuroinflammation, functional connectivity, and depressive symptoms in multiple sclerosis. Biol Psychiatry.

[59] Manning KJ (2016). Hippocampal neuroinflammation and depression: relevance to multiple sclerosis and other neuropsychiatric illnesses. Biol Psychiatry.

[60] Peruga I, Hartwig S, Thone J, Hovemann B, Gold R, Juckel G, Linker RA (2011). Inflammation modulates anxiety in an animal model of multiple sclerosis. Behav Brain Res.

[61] Haji N, Mandolesi G, Gentile A, Sacchetti L, Fresegna D, Rossi S, Musella A, Sepman H, Motta C, Studer V, De Chiara V, Bernardi G, Strata P, Centonze D (2012). TNF-alpha-mediated anxiety in a mouse model of multiple sclerosis. Exp Neurol.

[62] Acharjee S, Nayani N, Tsutsui M, Hill MN, Ousman SS, Pittman QJ (2013). Altered cognitive-emotional behavior in early experimental autoimmune encephalitis–cytokine and hormonal correlates. Brain Behav Immun.

[63] Kalinichenko LS, Kornhuber J, Muller CP (2019). Individual differences in inflammatory and oxidative mechanisms of stress-related mood disorders. Front Neuroendocrinol.

[64] Chen HC, Lee JK, Yip T, Sernia C, Lavidis NA, Spiers JG (2019). Sub-acute restraint stress progressively increases oxidative/nitrosative stress and inflammatory markers while transiently upregulating antioxidant gene expression in the rat hippocampus. Free Radic Biol Med.

[65] Cornell J, Salinas S, Huang HY, Zhou M (2022). Microglia regulation of synaptic plasticity and learning and memory. Neural Regen Res.

[66] Sun Y, Qu Y, Zhu J (2021). The relationship between inflammation and post-traumatic stress disorder. Front Psychiatry.

[67] Davalos D, Grutzendler J, Yang G, Kim JV, Zuo Y, Jung S, Littman DR, Dustin ML, Gan WB (2005). ATP mediates rapid microglial response to local brain injury in vivo. Nat Neurosci.

[68] Kreutzberg GW (1996). Microglia: a sensor for pathological events in the CNS. Trends Neurosci.

[69] Molfino A, Gioia G, Rossi Fanelli F, Laviano A (2015). Contribution of neuroinflammation to the pathogenesis of cancer cachexia. Mediators Inflamm.

[70] Qin C, Zhou LQ, Ma XT, Hu ZW, Yang S, Chen M, Bosco DB, Wu LJ, Tian DS (2019). Dual functions of microglia in ischemic stroke. Neurosci Bull.

[71] Norden DM, Trojanowski PJ, Villanueva E, Navarro E, Godbout JP (2016). Sequential activation of microglia and astrocyte cytokine expression precedes increased Iba-1 or GFAP immunoreactivity following systemic immune challenge. Glia.

[72] de Baaij JH, Hoenderop JG, Bindels RJ (2015). Magnesium in man: implications for health and disease. Physiol Rev.

[73] Brandao K, Deason-Towne F, Perraud AL, Schmitz C (2013). The role of Mg2+ in immune cells. Immunol Res.

[74] Yu X, Guan PP, Zhu D, Liang YY, Wang T, Wang ZY, Wang P (2018). Magnesium ions inhibit the expression of tumor necrosis factor alpha and the activity of gamma-secretase in a beta-amyloid protein-dependent mechanism in APP/PS1 transgenic mice. Front Mol Neurosci.

[75] Wang P, Yu X, Guan PP, Guo JW, Wang Y, Zhang Y, Zhao H, Wang ZY (2017). Magnesium ion influx reduces neuroinflammation in Abeta precursor protein/Presenilin 1 transgenic mice by suppressing the expression of interleukin-1beta. Cell Mol Immunol.

[76] Li X, Li L, Tao L, Zheng H, Sun M, Chen Y, Chen Y, Yang Y (2021). Magnesium sulfate prophylaxis attenuates the postpartum effects of preeclampsia by promoting M2 macrophage polarization. Hypertens Res.

[77] Gao F, Ding B, Zhou L, Gao X, Guo H, Xu H (2013). Magnesium sulfate provides neuroprotection in lipopolysaccharide-activated primary microglia by inhibiting NF-kappaB pathway. J Surg Res.

[78] Ning K, Liu WW, Huang JL, Lu HT, Sun XJ (2018). Effects of hydrogen on polarization of macrophages and microglia in a stroke model. Med Gas Res.

[79] Chu X, Cao L, Yu Z, Xin D, Li T, Ma W, Zhou X, Chen W, Liu D, Wang Z (2019). Hydrogen-rich saline promotes microglia M2 polarization and complement-mediated synapse loss to restore behavioral deficits following hypoxia-ischemic in neonatal mice via AMPK activation. J Neuroinflammation.

