# Supplementary material for: Nanoparticulate MgH2 ameliorates anxiety/depression-like behaviors in a mouse model of multiple sclerosis by regulating microglial polarization and oxidative stress
Source: J Neuroinflammation. 2023 Jan 30;20:16. doi: 10.1186/s12974-023-02696-y (PMC9885636; doi:10.1186/s12974-023-02696-y)
Supplement: Supplementary file 1 — Additional file 1: Figure S1. Confirming the concentration of MgH2 in vitro. Cell viability of BV2 in different concentrations of MgH2 (F = 16.26), n = at least 5 repeats per group. Data are presented as mean ± SEM. Figure S2. MgH2 treatment promotes the maturation of OPCs in EAE mice. (A) Representative anti-Ki67 (gray), anti-PDGFRα (red), and anti-Sox10 (green) immunofluorescence of the CC. (B) Quantitative analysis of PDGFRα+Sox10+ cells/mm2 (F = 0.4957) and Ki67+PDGFRα+Sox10+ cells/mm2 (F = 0.4059), n = 3 mice per group. (C) Representative anti-CC1 (red) and anti-Sox10 (green) immunofluorescence of the CC. (D) Quantitative analysis of CC1+Sox10+ cells/mm2 (F = 2.7514), n = 3 mice per group. Data are presented as mean ± SEM. Figure S3. Myelination and axons do not alter after 24-h restraint stress and MgH2 treatment. (A) Representative anti-MBP (red), anti-NF200 (green), and anti-SMI32 (purple) immunofluorescence of the CC. (B, C) Quantitative analysis of the MFI of MBP (F = 0.092) and NF200 (F = 0.0109), n = 3 mice per group. (D) Representative anti-Ki67 (gray), anti-PDGFRα (red), and anti-Sox10 (green) immunofluorescence of the CC. (E) Quantitative analysis of PDGFRα+Sox10+ cells/mm2 (F = 0.0323) and Ki67+PDGFRα+Sox10+ cells/mm2 (F = 0.1672), n = 3 mice per group. (F) Representative anti-CC1 (red) and anti-Sox10 (green) immunofluorescence of the CC. (G) Quantitative analysis of CC1+Sox10+ cells/mm2 (F = 0.1775), n = 3 mice per group. Data are presented as mean ± SEM. Figure S4. MgH2 treatment has no effect on the long-term proliferation of OPCs after 24-h restraint stress. (A) Schematic diagram displaying the cell proliferation test in 4 different groups. (B) Representative anti-BrdU (red), anti-Olig2 (green) and anti-Sox10 (purple) immunofluorescence of the CC. (C) Quantitative analysis of BrdU+Olig2+Sox10+ cells/mm2 (F = 0.1466), n = 3 mice per group. Data are presented as mean ± SEM. Figure S5. Number of infiltrating cells is reduced by MgH2 treatment after [file 12974_2023_2696_MOESM1_ESM.docx]

Additional Fig 1


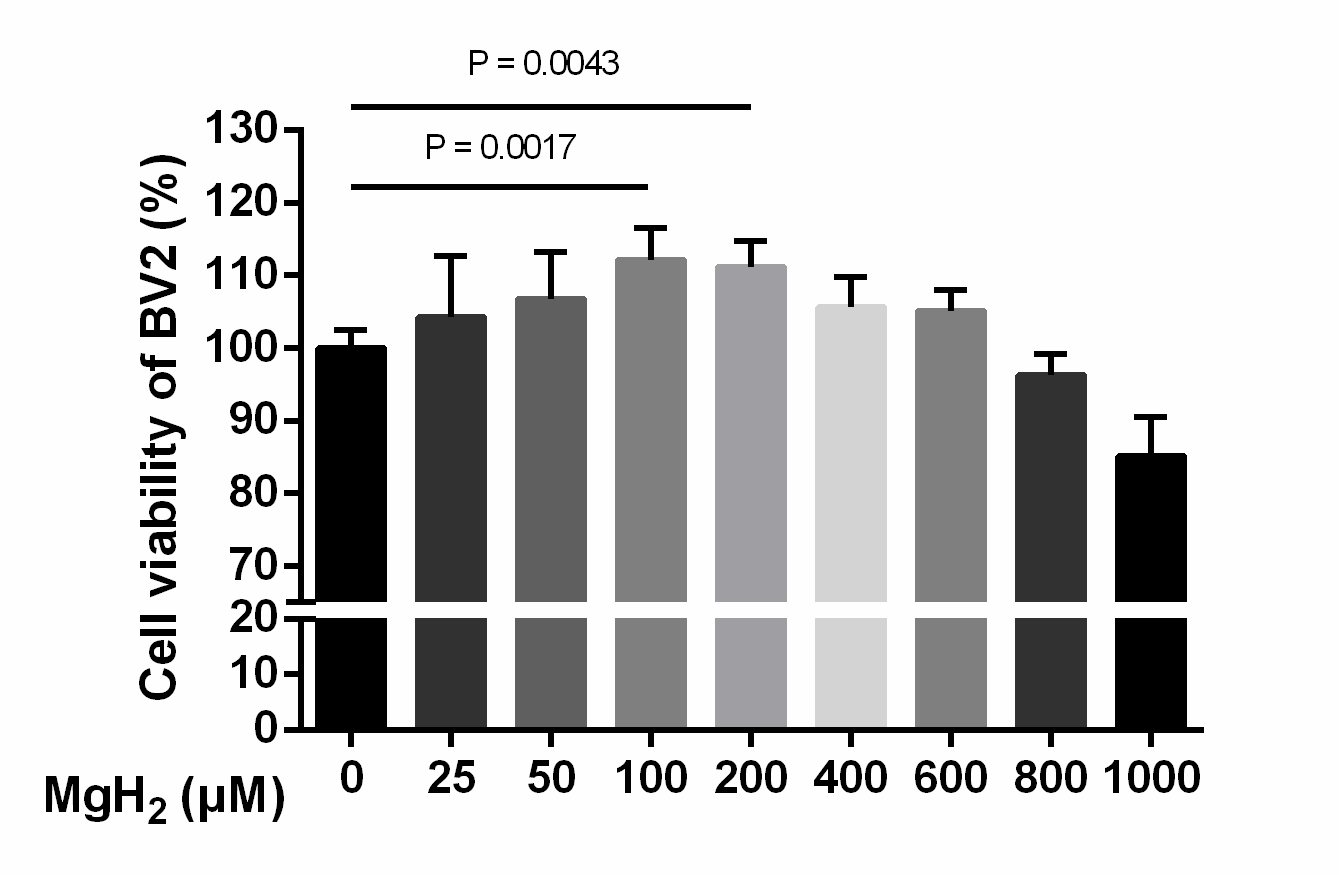


Additional Fig 2


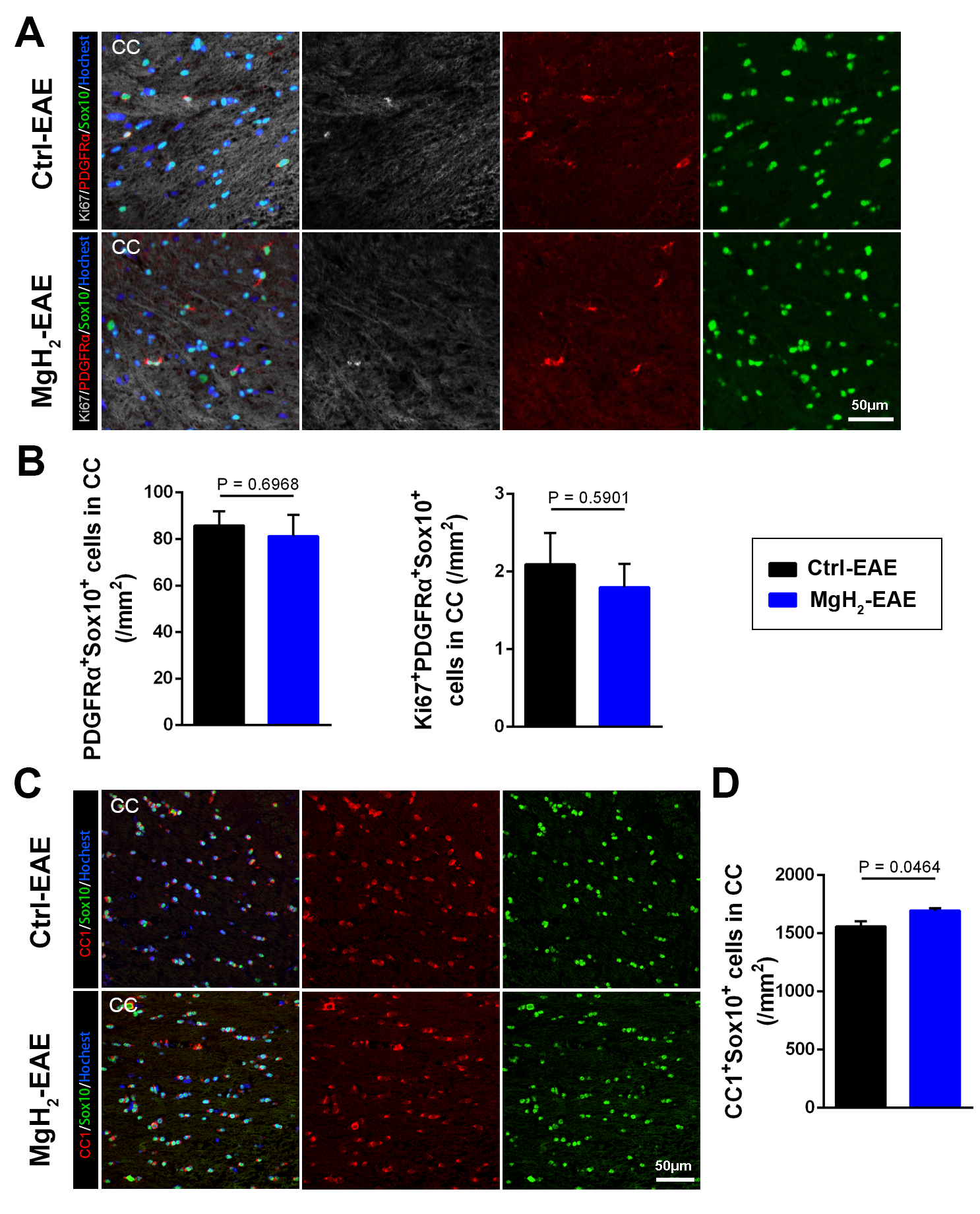


Additional Fig 3


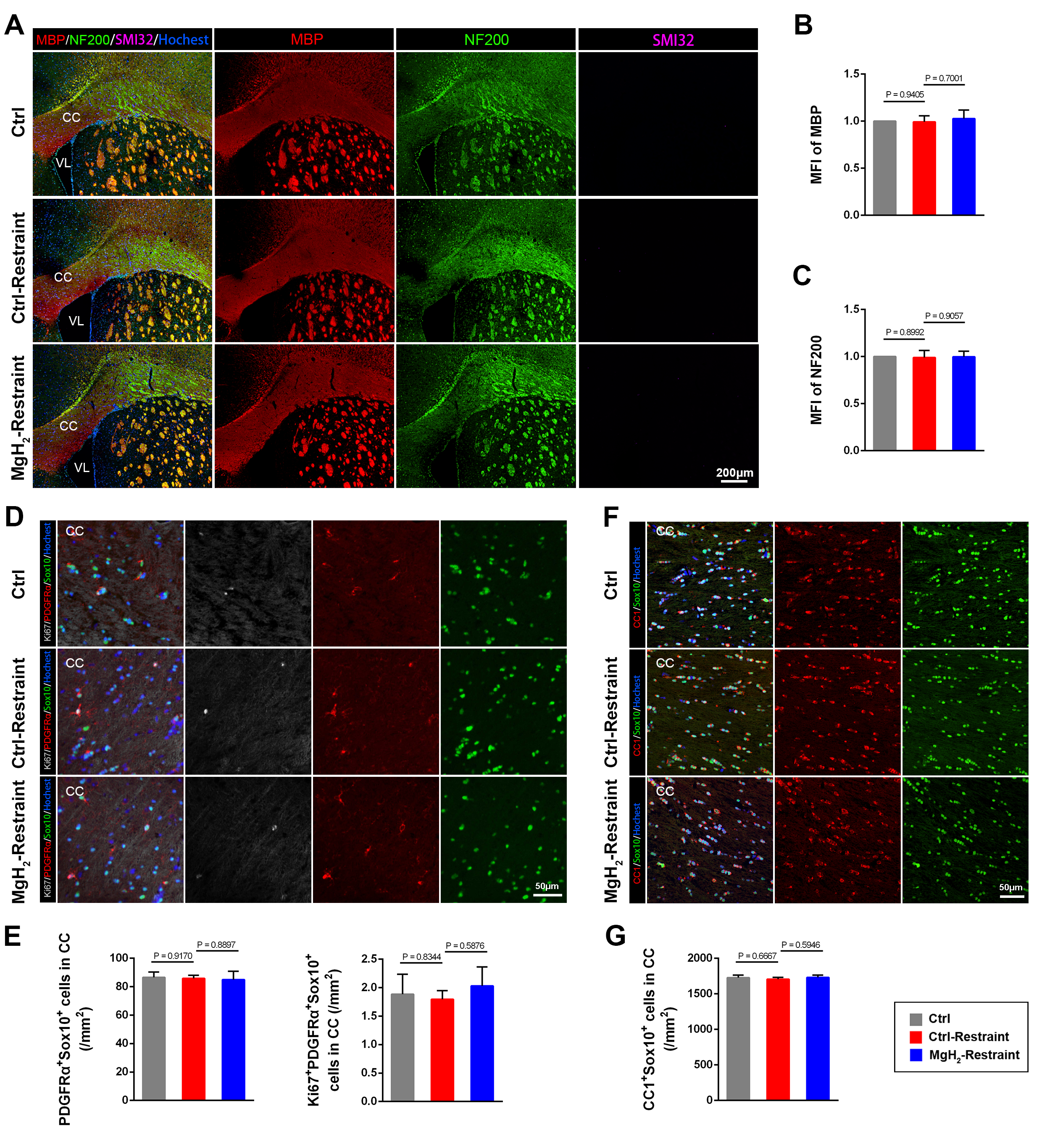


Additional Fig 4


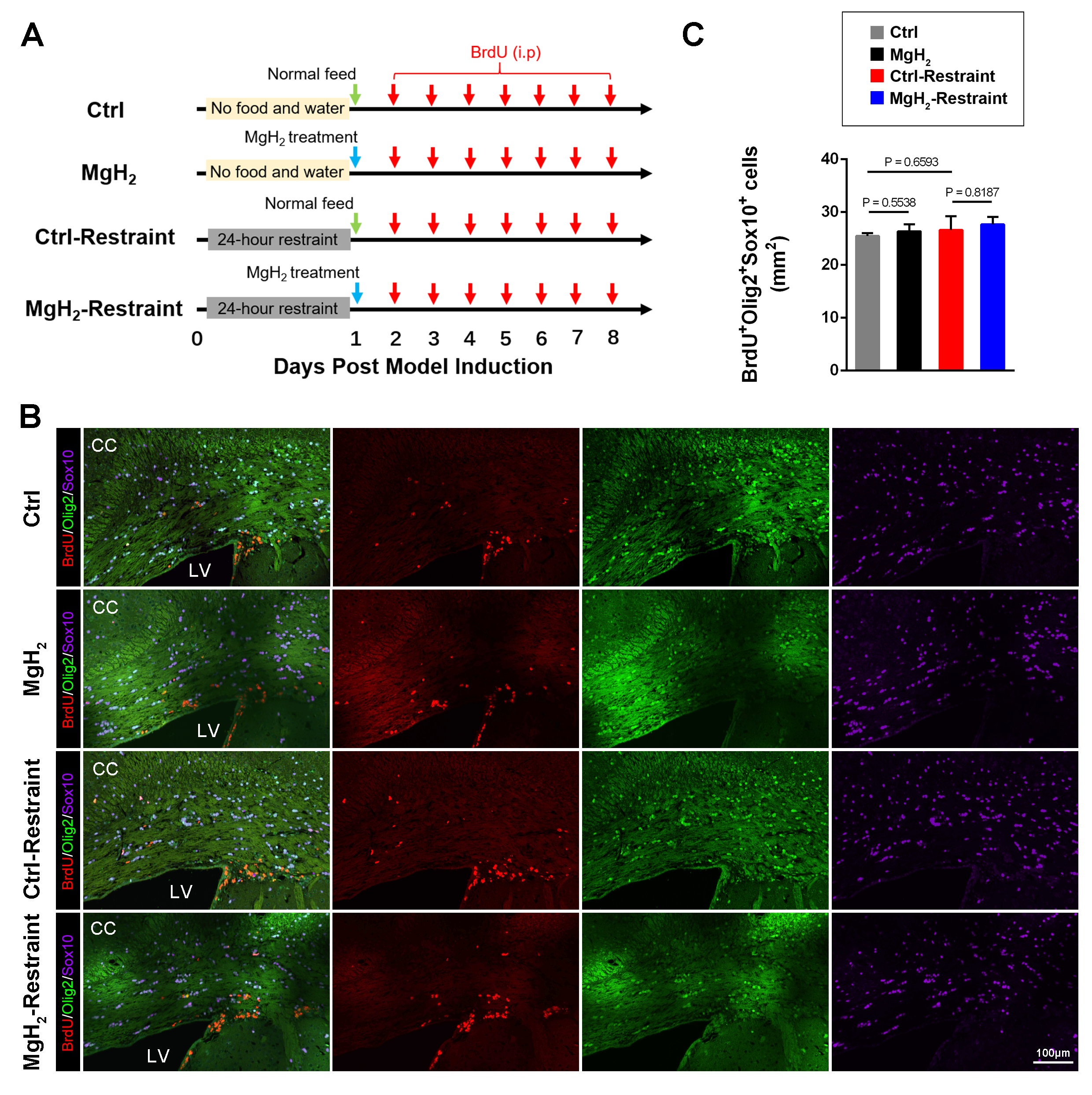


Additional Fig 5


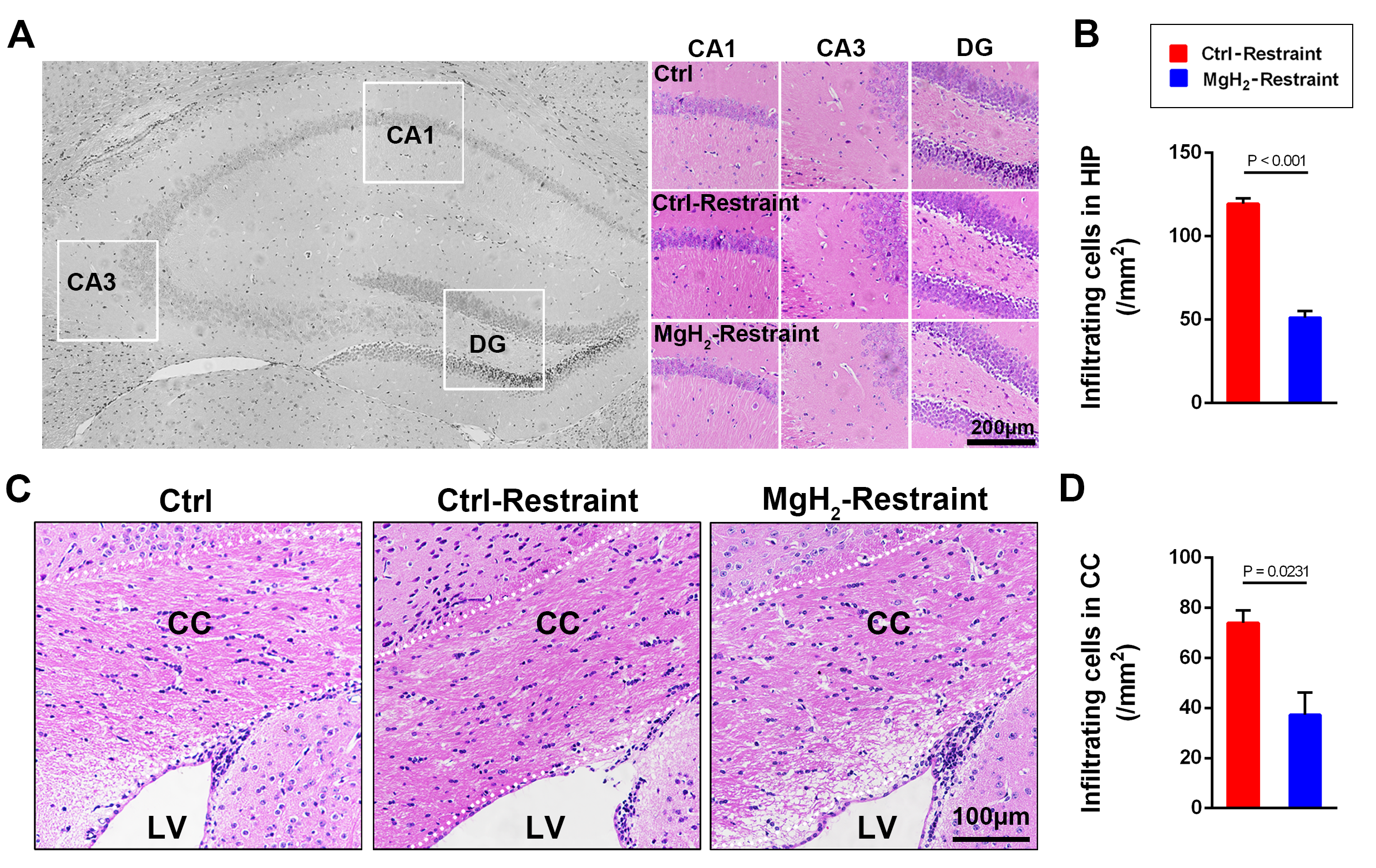


Additional Fig 6


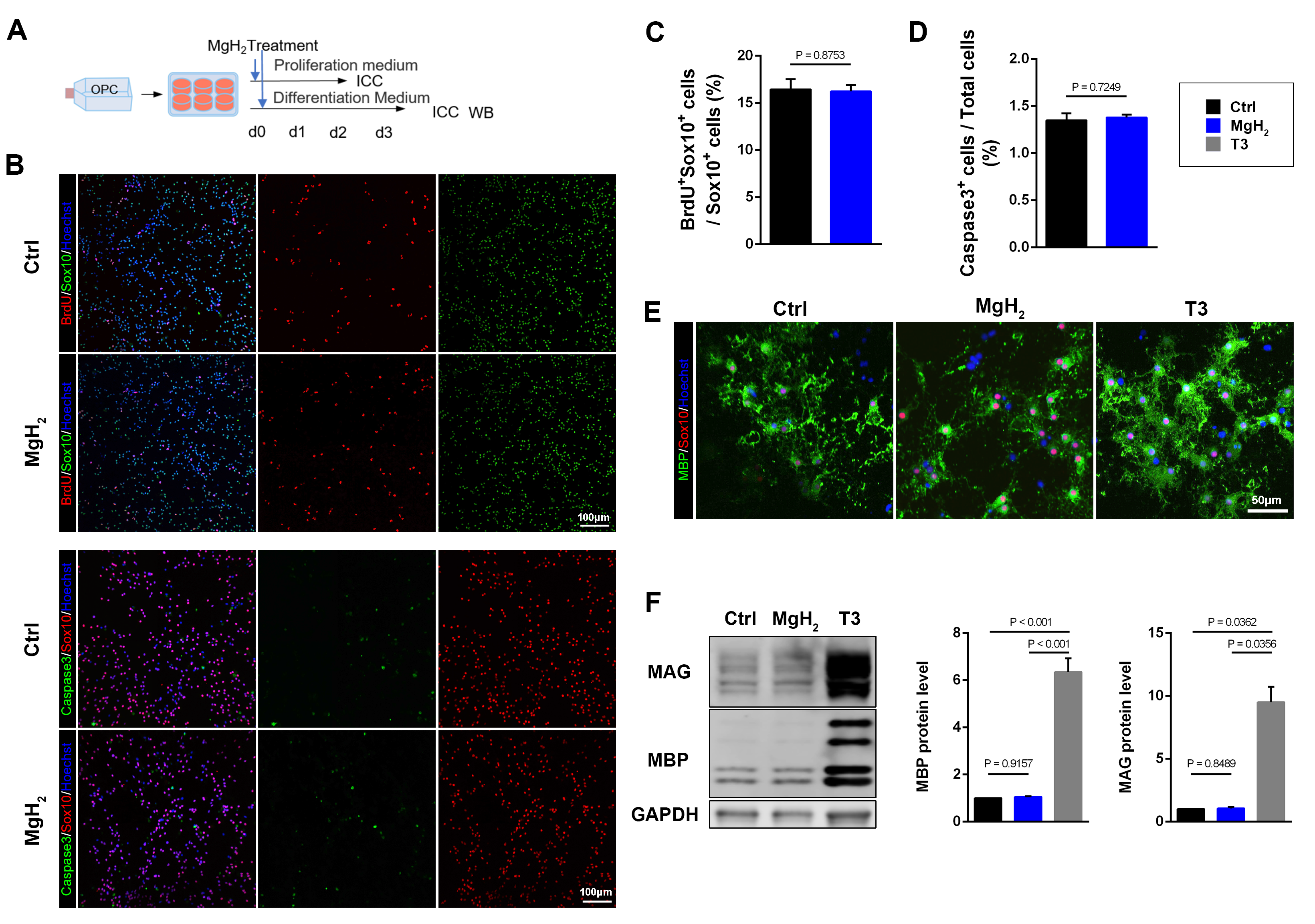


Additional Fig 6 (full and uncropped blot images)


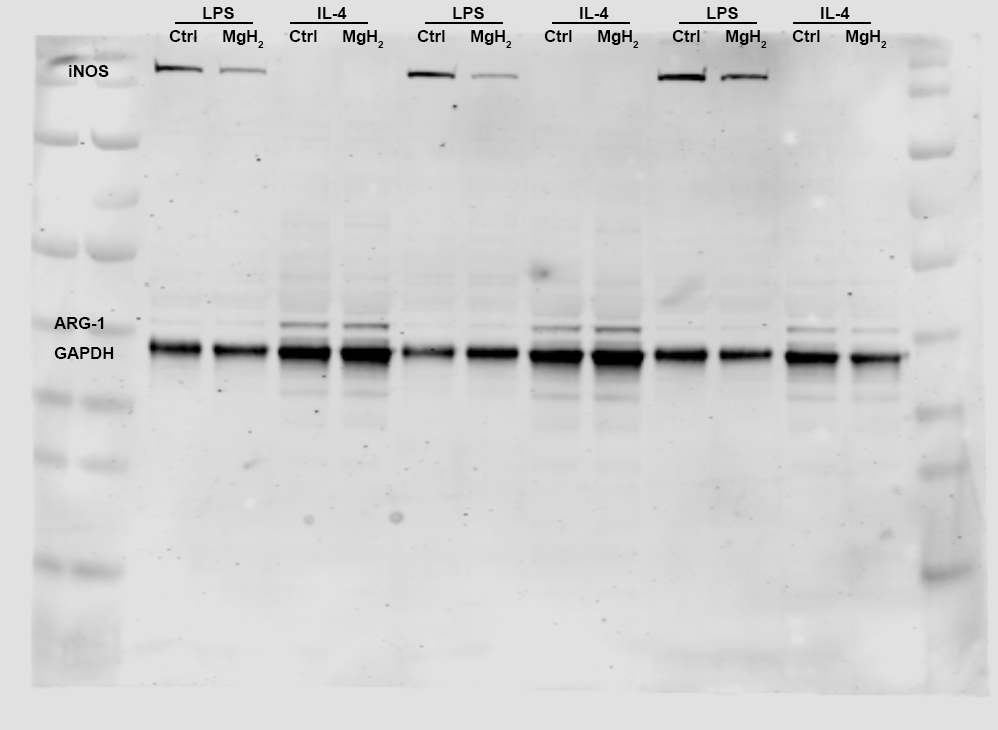


This original image corresponds to Fig 6C.


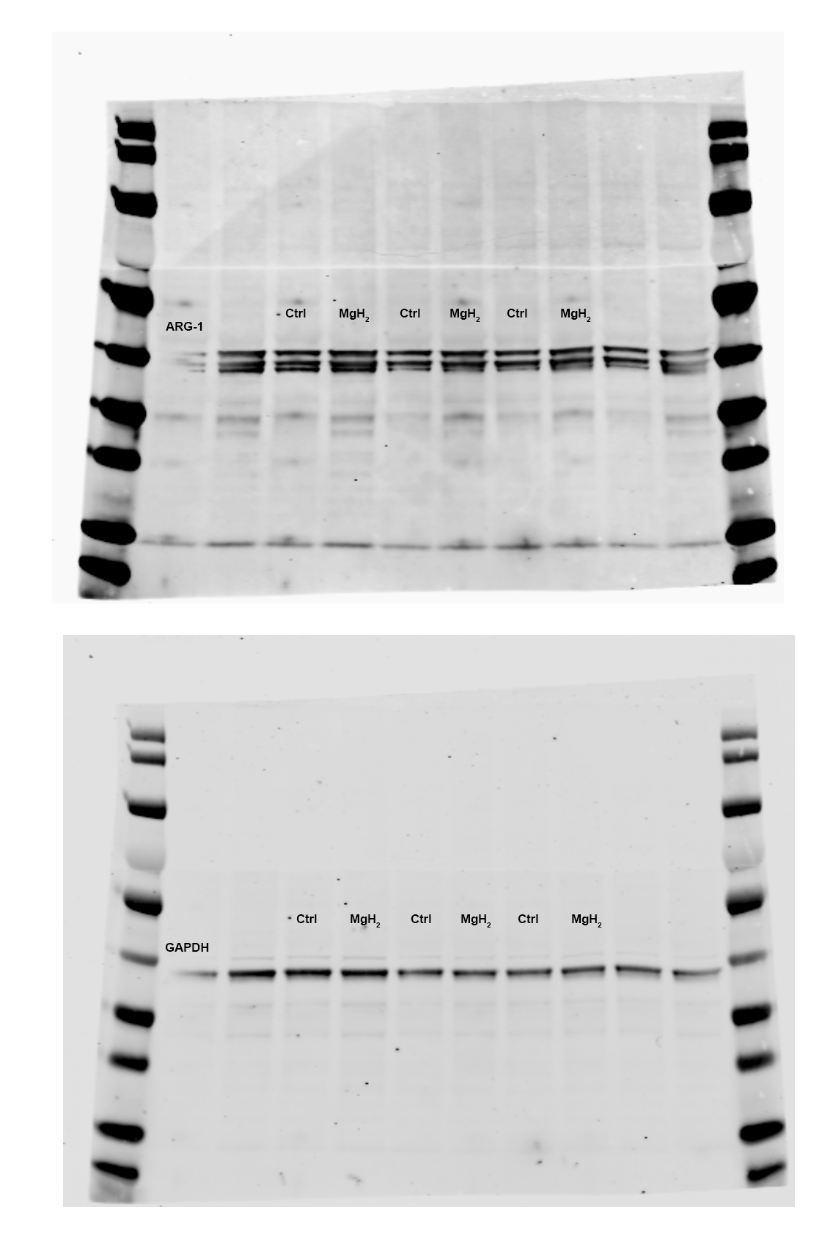


This original image corresponds to Fig 6E.


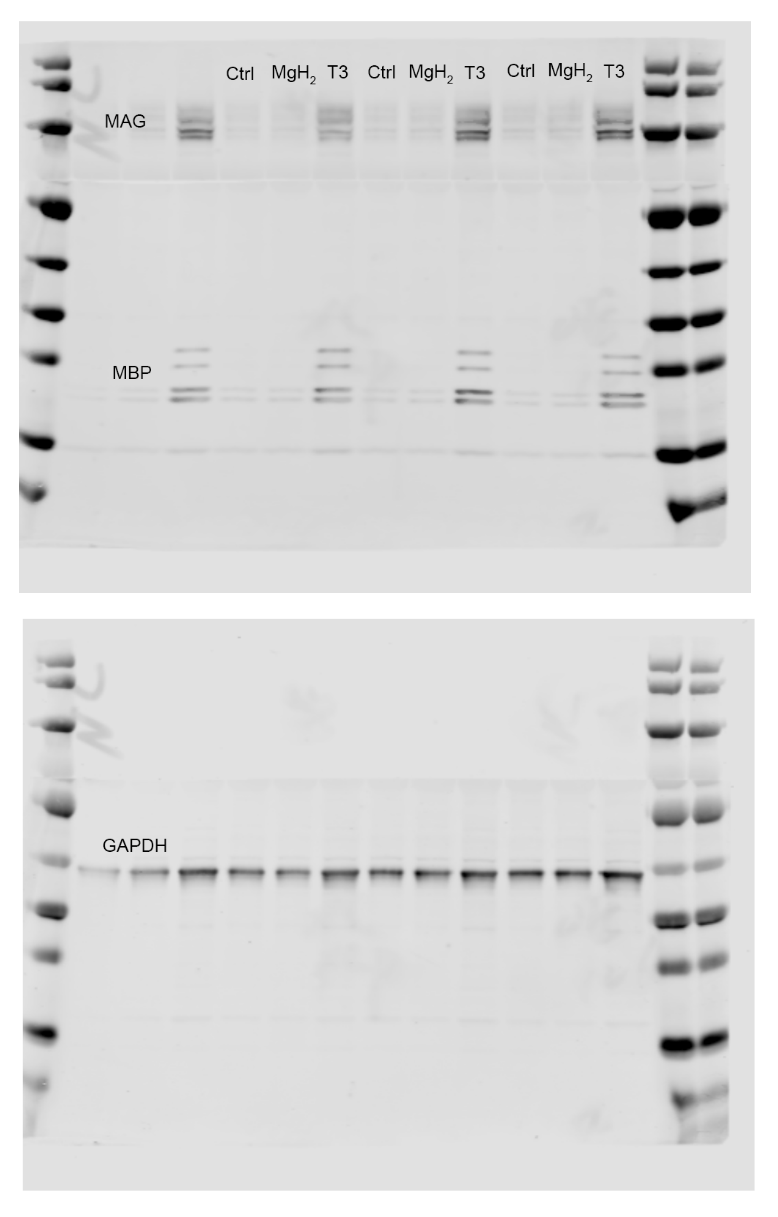


This original image corresponds to Additional Fig 5F.
